# Maternal cold exposure improves offspring metabolic health via a milk lithocholic acid–microbiota–Th17 axis

**DOI:** 10.1038/s41522-026-01092-7

**Published:** 2026-07-09

**Authors:** Xuejie Han, Hui Yu, Qianhui Gao, Dongze Li, Linwei Zhang, Qi Kang, Chaowen Sui, Qian Zhao, Xuanrui Ji, Hanwen Liu, Yuchen Wang, Changming Zhao, Shuting Li, Li Sun, Yun Zhang, Yue Li

**Affiliations:** 1 Department of Cardiology, the First Affiliated Hospital, Harbin Medical University, Harbin, China; 2 State Key Laboratory of Frigid Zone Cardiovascular Disease, Harbin Medical University, Harbin, Heilongjiang China; 3 Key Laboratory of Cardiac Diseases and Heart Failure, Harbin Medical University, Harbin, China; 4 Arongqi People’s Hospital, Arong Banner, Hulunbuir, Inner Mongolia China; 5 Department of Cardiology, The First Affiliated Hospital of USTC, Division of Life Sciences and Medicine, University of Science and Technology of China, Hefei, Anhui China

**Keywords:** Diseases, Endocrinology, Microbiology, Physiology

## Abstract

Metabolic diseases are rising with a trend toward earlier onset, yet effective preventive strategies remain limited. While cold exposure improves metabolic health in adults, its role during pregnancy in shaping offspring metabolic outcomes remains unknown. Herein, we demonstrate that maternal cold exposure in early pregnancy markedly improved offspring glucose tolerance, insulin sensitivity, and hepatic lipid metabolism when challenged with a Western diet, and the benefits persisted into late adulthood. Transcriptomic and immunophenotyping analyses revealed that offspring with cold-exposed dams exhibited suppressed Th17 activity and IL-17 signaling. Cross-fostering and metabolomics identified elevated lithocholic acid (LCA) in maternal milk as a critical mediator of these effects. LCA supplementation recapitulates these benefits through gut microbiota-dependent conversion to 3-oxo-LCA. Furthermore, *Clostridium scindens* supplementation enhanced 3-oxo-LCA production, suppressed Th17 responses, and alleviated diet-induced hepatic steatosis. Clinically, analysis of the UK Biobank cohort showed that winter conception was associated with a lower risk of metabolic dysfunction-associated steatotic liver disease in offspring. A similar association was observed in the CHARLS cohort in colder northern China. Together, these results identify a maternal cold–microbiota–bile acid–Th17 axis that programs offspring metabolic health and highlight microbial bile acid metabolism as a potential therapeutic target for metabolic diseases.

## Introduction

Metabolic diseases such as obesity, diabetes, and metabolic dysfunction-associated steatotic liver disease (MASLD)^1^ are rising at an alarming global pace^2–6^, driven in large part by the increasing prevalence of unhealthy dietary patterns, such as the Western diet. Strikingly, these conditions, once considered to emerge primarily in adulthood, are now frequently observed in childhood^7–10^, reflecting not only an earlier onset but also a concerning transgenerational shift in metabolic risk. This accelerating trend highlights the urgent need to identify modifiable environmental and nutritional factors in early life and to implement effective preventive strategies aimed at altering disease trajectories before they become irreversible.

The developmental origins of health and disease (DOHaD) framework posits that environmental exposures during critical windows of development, particularly the prenatal and perinatal periods, can exert lasting effects on offspring physiology^11–14^. A growing body of evidence supports this model, showing that maternal exposure to endocrine-disrupting chemicals (e.g., bisphenol analogs), microplastics, or suboptimal diets can predispose offspring to metabolic dysfunction in adulthood^15–18^. Conversely, beneficial maternal interventions—such as exercise or consumption of high-fiber or malate-enriched diets—have been shown to improve glucose tolerance, reduce hepatic lipid accumulation, and promote systemic metabolic health in offspring^19–24^. Among environmental factors, cold exposure represents a unique and underexplored stimulus. Cold activates thermogenic and lipid metabolic pathways in brown adipose tissue and enhances glutamine metabolism^25,26^. While adult cold exposure is known to affect energy balance and immune signaling, whether maternal cold exposure during pregnancy can exert long-lasting metabolic effects on the offspring remains largely unknown.

Bile acids (BAs) have emerged as key metabolic regulators that bridge host metabolism with immune responses^27^. Beyond their role in dietary lipid absorption, BAs function as signaling molecules that influence glucose homeostasis, lipid metabolism, and energy expenditure^28–32^. Primary BAs synthesized in the liver are further transformed by gut microbiota into secondary BAs, such as lithocholic acid (LCA), which have bioactive roles in immunity and metabolism. LCA is one of the most abundant secondary bile acids. It was reported that LCA conferred anti-ageing benefits by regulating metabolism in an AMP-activated protein kinase-dependent manner^33^. LCA and its bacterial metabolites—3-oxo-lithocholic acid (3-oxo-LCA) and isoalloLCA—have been shown to modulate CD4^+^ T-cell differentiation, suppressing proinflammatory Th17 responses and promoting regulatory T cells through nuclear hormone receptor modulation^34,35^. Given the emerging role of Th17-mediated inflammation in the pathogenesis of metabolic disorders such as MASLD^36,37^, these microbial metabolites represent promising targets for immunometabolic regulation.

In this study, we demonstrate that maternal cold exposure during early pregnancy confers long-term metabolic benefits to offspring challenged with a Western diet. Mechanistically, cold exposure increased LCA levels in maternal milk, which were subsequently converted by the offspring gut microbiota into 3-oxo-LCA. This metabolite suppressed Th17 cell differentiation and hepatic IL-17 signaling, thereby attenuating inflammation-driven lipid accumulation in the liver. Supplementation with *Clostridium scindens*, a bile acid–metabolizing bacterium, or 3-oxo-LCA reproduced these protective effects in offspring. Furthermore, analyses of the UK Biobank and CHARLS cohorts showed that winter conception was associated with a lower risk of MASLD in offspring. Together, these findings uncover a previously unrecognized bile acid–microbiota–Th17 axis linking maternal environmental exposure to offspring metabolic programming, suggesting that microbiota–bile acid–immune interactions could be targeted to prevent metabolic dysfunction.

## Results

### Maternal cold exposure during early pregnancy confers metabolic protection in offspring challenged with a Western diet

**Fig. 1:**
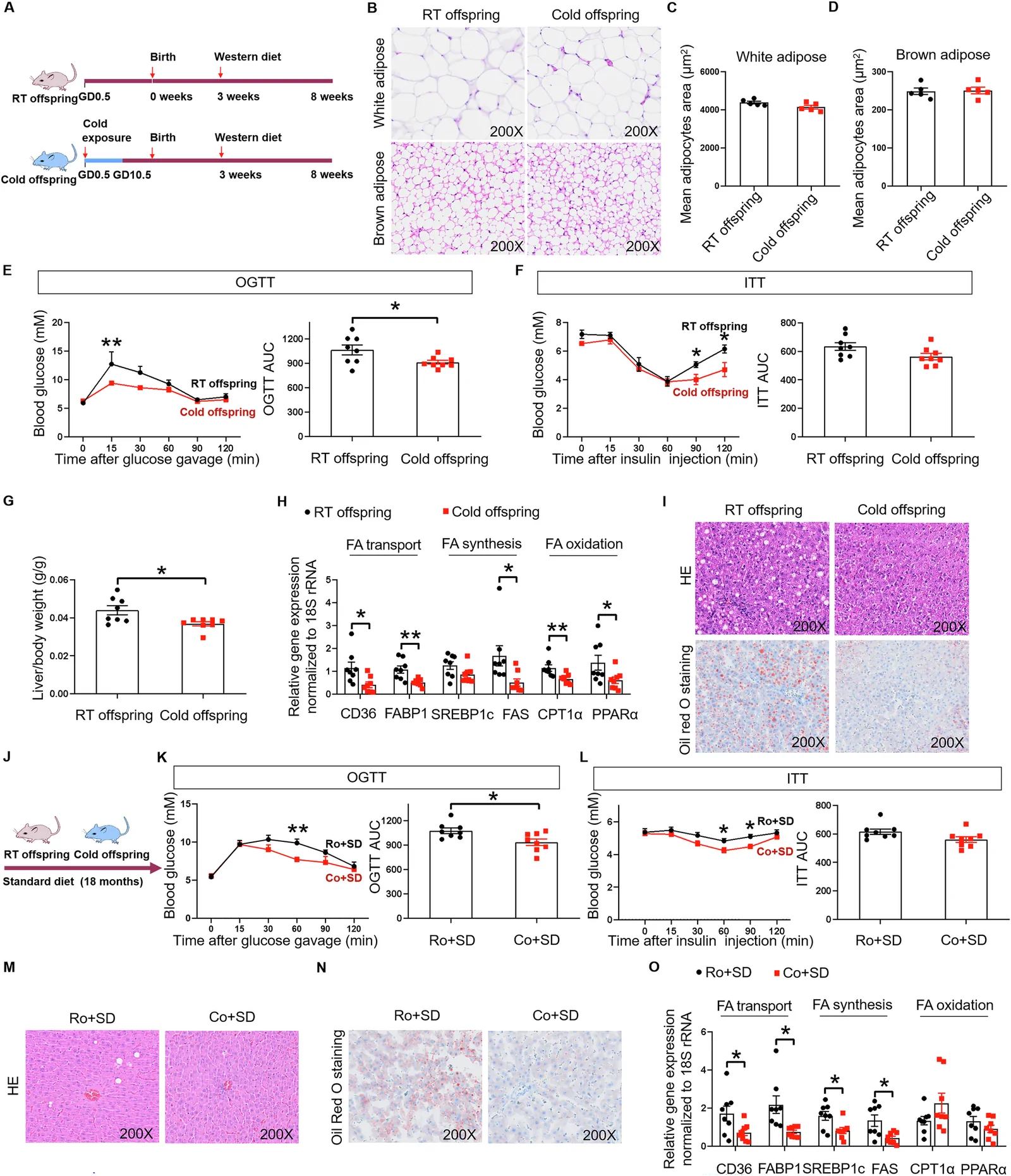
Maternal cold exposure protects against Western diet-induced glucose intolerance and hepatic steatosis in offspring. **A** Schematic diagram showing the establishment of a rat model of maternal cold exposure during pregnancy. Pregnant rats were exposed to room temperature (RT, 25 ± 1 °C) or cold exposure (4 ± 1 °C) for 10 days. Their offspring were referred to as RT offspring and Cold offspring, respectively, and all were raised under room temperature (RT) conditions after birth. After weaning at 3 weeks of age, the offspring were fed a Western diet for 5 weeks. **B** Representative images of H&E staining of white adipose tissues and brown adipose tissues from 8-week-old male RT and Cold offspring (200× magnification). Quantification of adipocyte size in white (**C**) and brown (**D**) adipose tissues (*n* = 5 per group). **E** Oral Glucose Tolerance Test (OGTT) in 8-week-old male RT and Cold offspring, with blood glucose curves and corresponding area under the curve (AUC) (*n* = 8 per group). **F** Insulin Tolerance Test (ITT) in 8-week-old male RT and Cold offspring, showing blood glucose levels over time and the AUC (*n* = 8 per group). **G** Liver-to-body weight ratio in 8-week-old male RT and Cold offspring (*n* = 8 per group). **H** The relative gene expression of fatty acid (FA) transport, synthesis, and oxidation pathways in 8-week-old male RT and Cold offspring (*n* = 8 per group). **I** Representative images for H&E and Oil Red O staining of liver tissues in male RT and Cold offspring (*n* = 8 per group) (200× magnification). **J** Schematic diagram: male RT and Cold offspring rats were raised at room temperature after birth, and were fed a standard diet post-weaning until 18 months of age. **K** Oral Glucose Tolerance Test (OGTT) in 18-month-old male RT and Cold offspring, with blood glucose curves and corresponding area under the curve (AUC) (*n* = 8 per group). **L** Insulin Tolerance Test (ITT) in 18-month-old male RT and Cold offspring, showing blood glucose levels over time and the AUC (*n* = 8 per group). **M** Representative images for H&E staining of liver tissues in 18-month-old male RT and Cold offspring (*n* = 8 per group) (200× magnification). **N** Representative images for Oil Red O staining of liver tissues in 18-month-old male RT and Cold offspring (200× magnification). **O** Gene expression of fatty acid transport, synthesis, and oxidation in 18-month-old male RT and Cold offspring. Data are presented as mean ± SEM and compared by Student’s *t*-test.

To investigate whether maternal cold exposure during pregnancy influences metabolic outcomes in offspring, pregnant rats were exposed to either room temperature (RT, 25 ± 1 °C) or cold conditions (Cold, 4 ± 1 °C) for 10 days during early gestation. Offspring from RT-exposed and cold-exposed dams (hereafter RT offspring and Cold offspring) were challenged with a Western diet post-weaning to unmask potential metabolic vulnerabilities (Fig. 1A). Maternal cold exposure did not affect reproductive outcomes, as birth rate and litter size were comparable between the two groups (Supplementary Fig. 1A, B). Maternal body weight during gestation was also not significantly different between RT and Cold-exposed dams (Supplementary Fig. 1C). Furthermore, male RT and Cold offspring exhibited similar body weight trajectories from birth to 8 weeks of age (Supplementary Fig. 2A, B). Similarly, no notable differences were observed between the two groups in the morphology or adipocyte size of white and brown adipose tissues at the end of a Western diet feeding (Fig. 1B–D). Despite these comparable growth and adipose tissue parameters, male Cold offspring displayed significantly improved glucose tolerance and insulin sensitivity compared with male RT offspring, as shown by oral glucose tolerance and insulin tolerance tests (Fig. 1E, F). In parallel, Cold offspring had a reduced liver-to-body weight ratio and liver weight (Fig. 1G and Supplementary Table1). Hepatic expression of genes involved in lipid uptake (CD36, FABP1), lipogenesis (FAS), and β-oxidation (CPT1α, PPARα) was significantly lower in Cold offspring than in RT offspring (Fig. 1H). Histological and quantitative analyses consistently showed reduced hepatic steatosis and lipid deposition in Cold offspring, as evidenced by decreased steatotic area, Oil Red O-positive area, and hepatic triglyceride level (Fig. 1I, Supplementary Fig. 2C–E). However, female RT and Cold offspring showed no obvious differences in hepatic phenotypes under the same experimental conditions (Supplementary Fig. 3A–H).

To assess whether these benefits persisted into late adulthood, parallel cohorts were maintained on standard chow until 18 months of age (Fig. 1J). Cold offspring retained superior glucose tolerance, insulin sensitivity, and reduced hepatic lipid content (Fig. 1K–N, Supplementary Fig. 4A–C), with persistently suppressed CD36, FABP1, and FAS expression (Fig. 1O). These findings indicate that transient maternal cold exposure during early pregnancy confers sustained metabolic benefits to offspring that persist into late life, even in the absence of postnatal dietary stress.

### Maternal cold exposure suppresses Th17 responses and hepatic inflammation in offspring

**Fig. 2:**
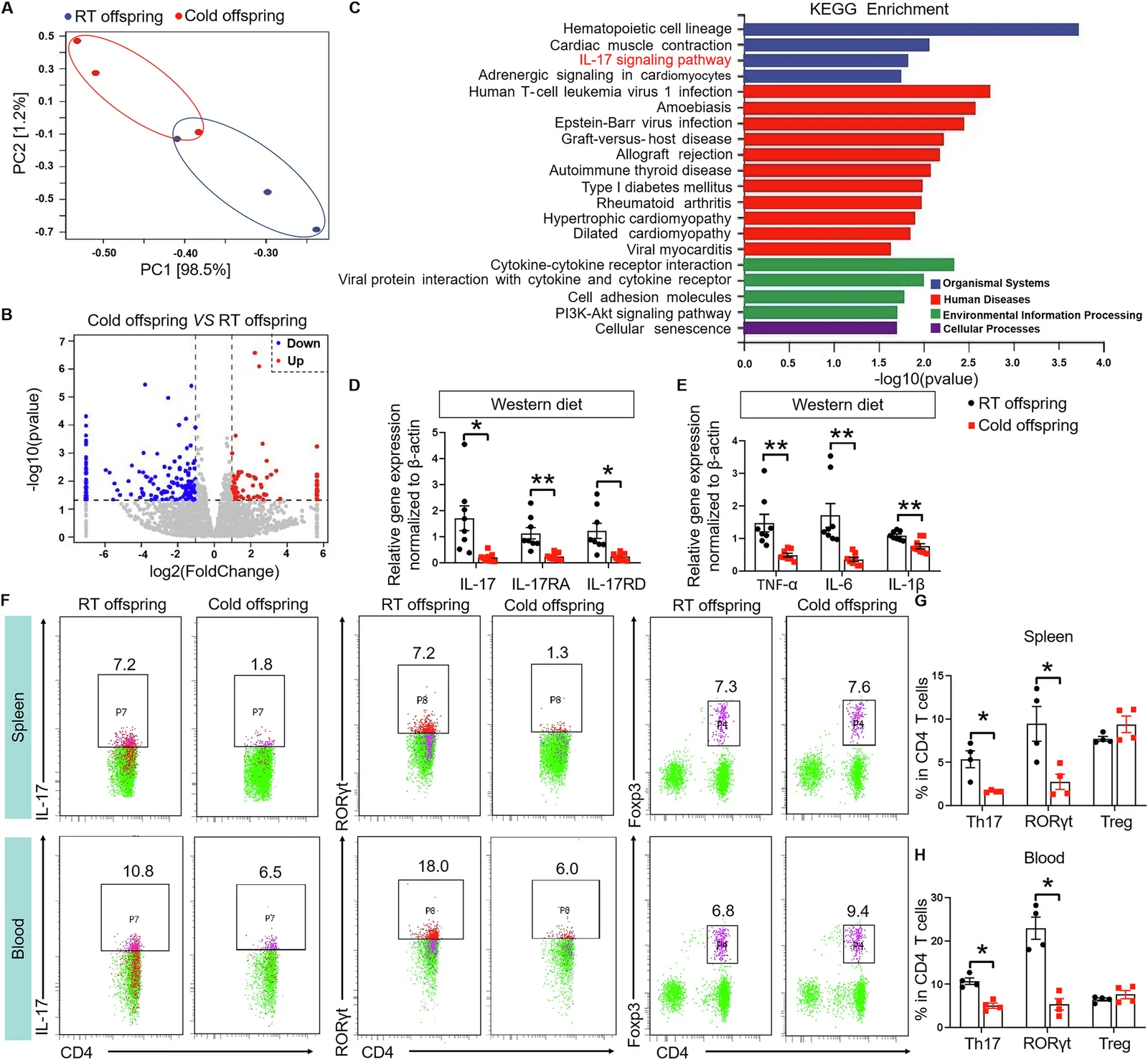
Maternal cold exposure during pregnancy reduces the Th17 cell proportion and IL-17 signaling in offspring. **A** Principal component analysis (PCA) based on liver transcriptome profiles reveals distinct clustering of male RT and Cold offspring (*n* = 3 per group). **B** Volcano plot of differentially expressed genes (DEGs) between male Cold and RT offspring, with upregulated genes in red and downregulated genes in blue. **C** Kyoto Encyclopedia of Genes and Genomes (KEGG) pathway enrichment analysis of DEGs, highlighting significant pathways including IL-17 signaling. **D** The relative mRNA expression of IL-17, IL-17RA, and IL-17RD in 8-week-old male RT and Cold offspring fed a Western diet (*n* = 8 per group). **E** The relative mRNA expression of TNF-α, IL-6, and IL-1β in 8-week-old male RT and Cold offspring fed a Western diet (*n* = 8 per group). **F** Flow cytometry analysis of Th17, RORγt, and Treg cells in the spleen and blood of male RT and Cold offspring. **G**, **H** Quantification of the percentage of Th17, RORγt, and Treg cells in the spleen and blood of male RT and Cold offspring (*n* = 4 per group). Data are presented as mean ± SEM and compared by Student’s *t*-test (**D** and **E**) or non-parametric Mann–Whitney U test (**G** and **H**).

To identify molecular mechanisms underlying the metabolic protection, we performed hepatic RNA-seq in 8-week-old Cold and RT offspring fed a Western diet. Differential gene expression analysis revealed significant transcriptomic changes between the groups (Fig. 2A, B). KEGG pathway enrichment analysis identified the IL-17 signaling pathway as one of the most significantly downregulated pathways in Cold offspring (Fig. 2C). qRT-PCR confirmed marked suppression of hepatic IL-17A, IL-17RA, and IL-17RD expression in Cold offspring (Fig. 2D). Additionally, we observed significant downregulation of other inflammatory cytokines, including TNF-α, IL-6, and IL-1β, in Cold offspring (Fig. 2E). Given the established role of Th17 cells in driving hepatic inflammation and metabolic dysfunction^36,38^, we quantified circulating and splenic Th17 populations by flow cytometry. Cold offspring exhibited significantly reduced Th17 cell frequencies in both peripheral blood and spleen, along with decreased expression of the lineage-defining transcription factor RORγt (Fig. 2F–H). Regulatory T-cell (Treg) populations remained unchanged. These findings suggest that maternal cold exposure is associated with reduced Th17 differentiation and improved hepatic metabolic parameters in offspring.

### Breast milk mediates the metabolic protection conferred by maternal cold exposure

**Fig. 3:**
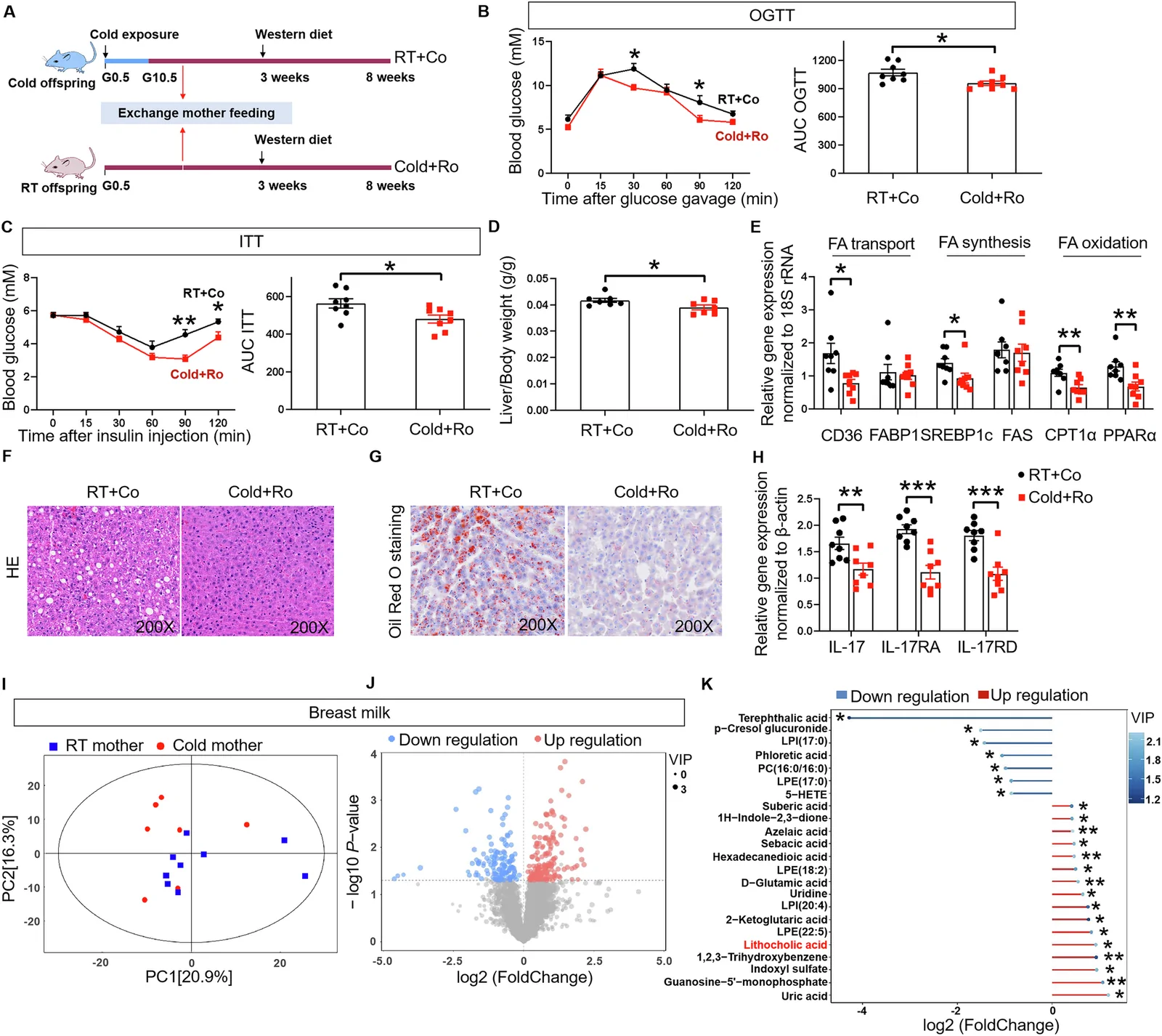
Maternal cold exposure confers metabolic benefits to offspring via breast milk. **A** Schematic diagram showing the workflow of cross-fostering experiments. Litters born to mothers exposed to cold during pregnancy were fostered by room-temperature (RT) mothers (RT + Co group), and litters born to RT mothers were fostered by cold-exposed mothers (Cold+Ro group) within 24 h after birth. Group nomenclature denotes the foster dam followed by the biological offspring (i.e., RT+Co refers to RT foster dams + Cold-origin offspring, indicating Cold-born offspring reared by room-temperature foster dams; Cold+Ro refers to Cold foster dams + RT-origin offspring, indicating RT-born offspring reared by Cold foster dams). After weaning at 3 weeks of age, both groups were fed a Western diet for 5 weeks. **B** Oral Glucose Tolerance Test (OGTT) in RT+Co and Cold+Ro offspring at 8 weeks, showing blood glucose levels over time and the corresponding area under the curve (AUC) (*n* = 8 per group). **C** Insulin Tolerance Test (ITT) in RT+Co and Cold+Ro offspring at 8 weeks, showing blood glucose levels over time and the AUC (*n* = 8 per group). **D** Liver-to-body weight ratio in RT+Co and Cold+Ro offspring at 8 weeks (*n* = 8 per group). **E** Gene expression analysis of fatty acid (FA) transport, synthesis, and oxidation in RT+Co and Cold+Ro offspring at 8 weeks (*n* = 8 per group). **F** Representative images for H&E staining of liver tissues in 8-week-old RT+Co and Cold+Ro offspring (200× magnification). **G** Representative images of Oil Red O staining of liver tissues in 8-week-old RT+Co and Cold+Ro offspring (200× magnification). **H** Gene expression of IL-17, IL-17RA, and IL-17RD in RT+Co and Cold+Ro offspring at 8 weeks (*n* = 8 per group). **I** Principal component analysis (PCA) of breast milk metabolite profiles from Cold mothers (*n* = 7) and RT mothers (*n* = 9). Milk metabolomics was performed on samples collected from all dams in this experimental batch that passed pre-analytical quality control. **J** Volcano plot of differentially regulated metabolites in breast milk from Cold mothers (*n* = 7) and RT mothers (*n* = 9). **K** VIP scores and log2 fold changes of the top upregulated and downregulated metabolites in breast milk from Cold mothers (*n* = 7) and RT mothers (*n* = 9). Data are presented as mean ± SEM and compared by Student’s *t*-test.

Given the critical role of breast milk in shaping offspring metabolism and immunity^39^, we performed cross-fostering experiments in which newborn pups were exchanged between Cold and RT dams immediately after birth. All offspring were weaned onto a Western diet and analyzed at 8 weeks of age (Fig. 3A). For clarity, cross-fostering groups were defined by the foster dam condition followed by the biological origin of the offspring. Specifically, RT + Co refers to RT foster dams nursing Cold-origin offspring, whereas Cold + Ro refers to Cold foster dams nursing RT-origin offspring. Strikingly, the Cold + Ro group exhibited significantly improved glucose tolerance and insulin sensitivity compared with the RT + Co group (Fig. 3B, C). These findings suggest that metabolic benefits are transmitted, at least in part, through postnatal factors, including lactation. Cold + Ro group also displayed reduced liver-to-body weight ratios and peritesticular fat weight (Fig. 3D and Supplementary Table1) and suppressed hepatic expression of genes governing lipid transport (CD36, FABP1), synthesis (FAS), and β-oxidation (CPT1α) (Fig. 3E), with markedly decreased hepatic steatosis and hepatic triglyceride levels (Fig. 3F–G, Supplementary Figure 5A–C). Immunologically, Cold + Ro group showed reduced hepatic IL-17 pathway gene expression (IL-17A, IL-17RA, IL-17RD; Fig. 3H), decreased circulating and splenic Th17 cell frequencies, and lower RORγt expression (Supplementary Fig. 5D–F). These findings support the possibility that maternal cold exposure is associated with metabolic and immunological benefits in offspring, potentially mediated in part by breast milk-associated factors.

### LCA in maternal milk is elevated by cold exposure and indirectly regulates Th17 cells via gut microbial metabolites

To identify specific breast milk components mediating the metabolic benefits of maternal cold exposure, we performed untargeted metabolomic profiling of milk from RT and Cold-exposed dams. Metabolomic analysis revealed significant compositional differences between the two groups (Fig. 3I, J). Notably, lithocholic acid (LCA), a secondary bile acid, was substantially enriched in the milk of cold-exposed dams compared to RT controls (Fig. 3K), a finding confirmed by ELISA (Supplementary Fig. 6).

**Fig. 4:**
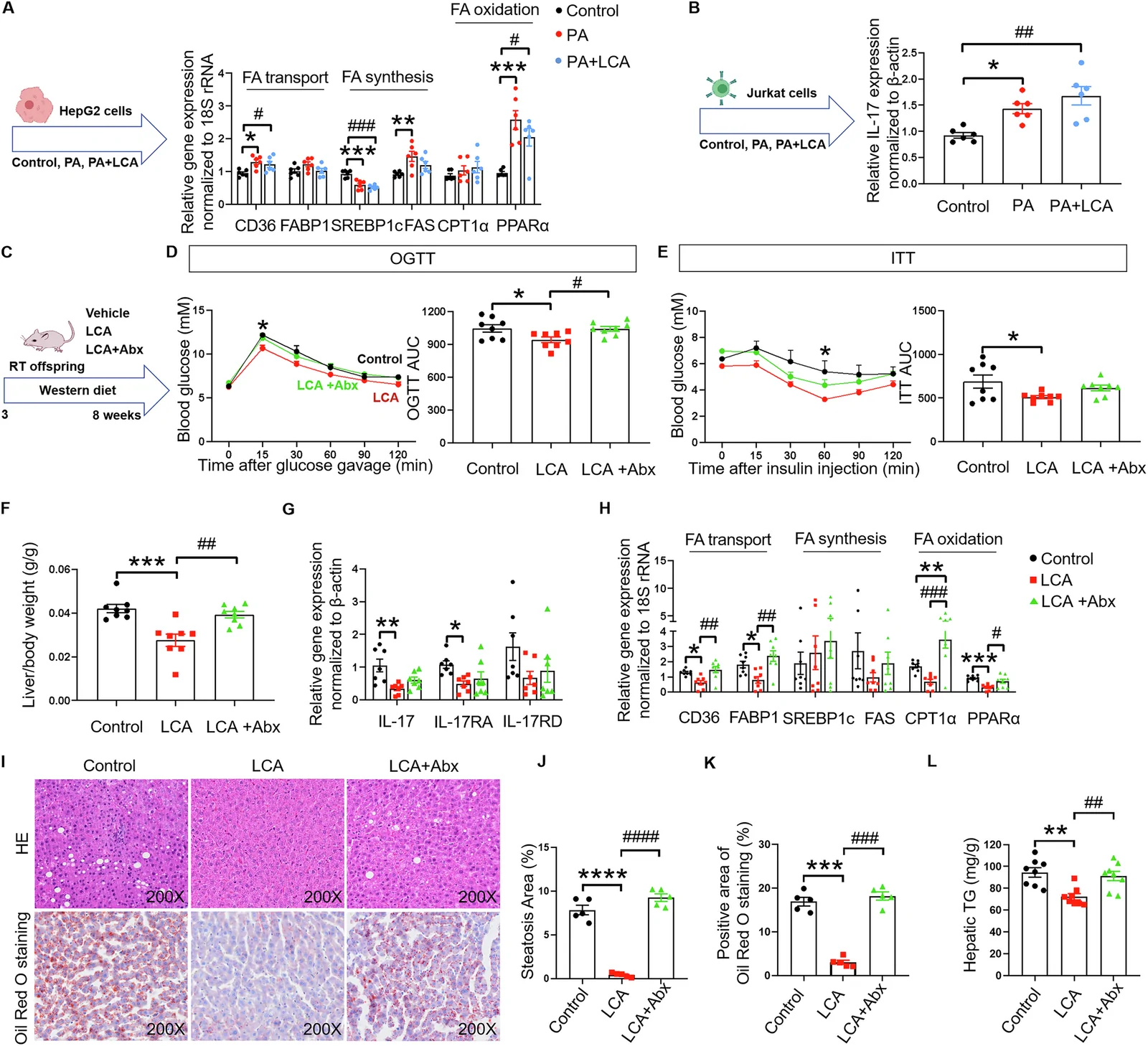
LCA exerts metabolic benefits depends on gut microbiota. **A** Gene expression of fatty acid transport, synthesis, and oxidation in HepG2 cells treated with control, PA, or PA + LCA (*n* = 6 per group). **B** The relative expression of IL-17 in Jurkat cells from Control, palmitic acid (PA), or PA + LCA groups (*n* = 6 per group). **C** Schematic diagram of the experimental design: Three-week-old RT offspring rats were randomly assigned to Control, LCA, and LCA+Abx groups. All groups were fed a Western diet for 5 weeks. **D** Oral Glucose Tolerance Test (OGTT) in Control, LCA, and LCA + Abx-treated offspring, showing blood glucose levels over time and the corresponding area under the curve (AUC) (*n* = 8 per group). **E** Insulin Tolerance Test (ITT) in Control, LCA, and LCA + Abx-treated offspring, showing blood glucose levels over time and the AUC (*n* = 8 per group). **F** Liver-to-body weight ratio in Control, LCA, and LCA + Abx-treated offspring at 8 weeks (*n* = 8 per group). **G** Gene expression of IL-17, IL-17RA, and IL-17RD in Control, LCA, and LCA + Abx-treated offspring (*n* = 8 per group). **H** Gene expression of fatty acid (FA) transport, synthesis, and oxidation in Control, LCA, and LCA + Abx-treated offspring (*n* = 8 per group). **I** Representative images for H&E staining and Oil Red O staining of liver tissues in rats (200× magnification). **J** Quantification of hepatic steatosis area by H&E staining in rats (*n* = 5 per group). **K** Quantification of lipid deposition by Oil Red O-positive area in rats (*n* = 5 per group). **L** Hepatic triglyceride (TG) content (*n* = 8 per group). Data are presented as mean ± SEM and compared by Student’s *t*-test.

To assess the functional relevance of LCA, we evaluated its effects in vitro using palmitic acid (PA) to induce lipid overload. In HepG2 cells, PA altered the expression of genes involved in fatty acid transport, synthesis, and oxidation compared with control. However, co-treatment with LCA failed to reverse these PA-induced transcriptional changes (Fig. 4A). In Jurkat T cells, PA significantly increased IL-17A expression compared with control, but co-treatment with LCA did not significantly attenuate PA-induced IL-17A expression (Fig. 4B). These results suggest that LCA does not exert direct regulatory effects on hepatocytes or T cells under lipid-overload conditions. Given that LCA is a gut microbiota-derived secondary bile acid subject to further bacterial metabolism^40,41^, we hypothesized that LCA-derived microbial metabolites, rather than LCA itself, might be responsible for suppressing Th17 responses. To test this hypothesis, we administered LCA or vehicle to rats for two weeks and subsequently collected fecal supernatants to stimulate Jurkat T cells (Supplementary Fig. 7A). Strikingly, fecal supernatants from LCA-treated animals potently suppressed PA-induced IL-17A expression in T cells compared with vehicle controls (Supplementary Fig. 7B). These findings indicate that LCA may act indirectly through gut microbial metabolism to regulate Th17 cell activity.

### LCA supplementation protects against Western diet-induced metabolic dysfunction via a gut microbiota-dependent mechanism

To determine whether LCA and its microbial metabolites confer metabolic protection in vivo, we divided RT offspring into three experimental groups: vehicle control, LCA supplementation, and LCA plus antibiotic co-treatment, all maintained on a Western diet (Fig. 4C). LCA supplementation significantly improved glucose tolerance and insulin sensitivity compared with vehicle-treated controls, while antibiotic co-treatment completely abrogated these metabolic improvements (Fig. 4D, E). Furthermore, LCA-treated offspring exhibited a reduced liver-to-body weight ratio and liver weight (Fig. 4F and Supplementary Table1). LCA treatment also significantly suppressed hepatic expression of IL-17A and IL-17RA (Fig. 4G). Consistently, LCA treatment downregulated hepatic expression of genes involved in lipid transport, synthesis, and β-oxidation (CD36, FABP1, SREBP1c, FAS, CPT1α, PPARα) (Fig. 4H), along with markedly decreased hepatic steatosis, lipid accumulation, and hepatic triglyceride levels (Fig. 4I–L). Notably, these hepatic improvements were abolished by antibiotic co-treatment. Together, these findings suggest that LCA may mediate metabolic protection through a gut microbiota-dependent mechanism associated with suppression of Th17 cell responses.

### 3-oxo-LCA, a gut microbiota-derived metabolite of lithocholic acid, mediates metabolic protection by suppressing Th17 responses

**Fig. 5:**
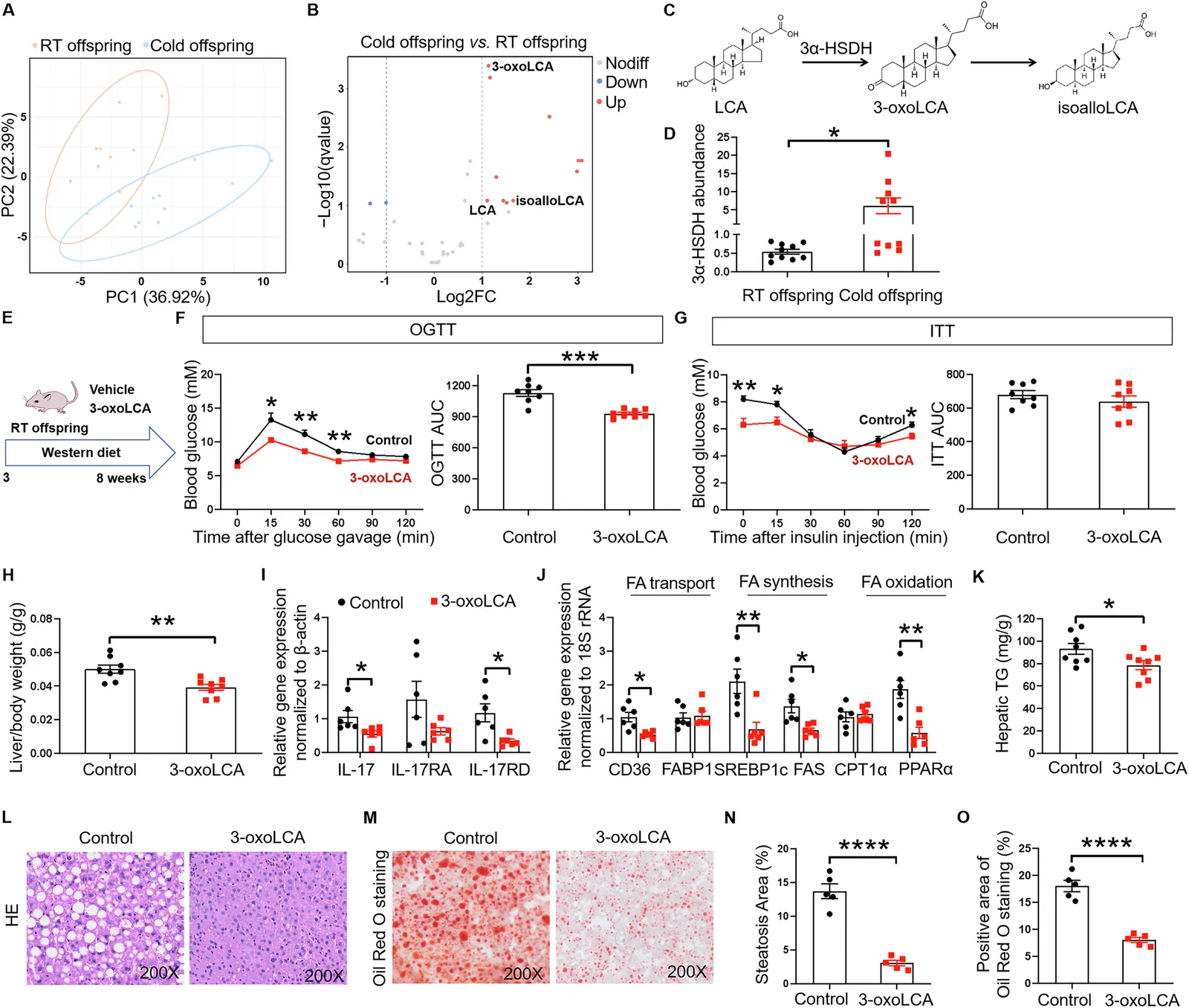
3-oxo-LCA improves Western diet-induced glucose dysregulation and hepatic steatosis in offspring. **A** Principal component analysis (PCA) of targeted plasma bile acid metabolomics data showing distinct clustering between RT and Cold offspring (*n* = 10 per group). **B** Volcano plot showing differentially expressed metabolites between Cold offspring and RT offspring. Red dots indicate significantly upregulated metabolites; blue dots indicate significantly downregulated metabolites. **C** Chemical structure of LCA, 3-oxo-LCA, and isoalloLCA, with the conversion of LCA to 3-oxo-LCA by 3α-HSDH. **D** Quantification of 3α-HSDH abundance in RT and Cold offspring (*n* = 10 per group). **E** Experimental timeline for 3-oxo-LCA treatment in RT offspring with a Western diet for 5 weeks. **F** Oral Glucose Tolerance Test (OGTT) in Control and 3-oxo-LCA-treated offspring, showing blood glucose levels over time and the corresponding area under the curve (AUC) (*n* = 8 per group). **G** Insulin Tolerance Test (ITT) in Control and 3-oxo-LCA-treated offspring, showing blood glucose levels over time and the AUC (*n* = 8 per group). **H** Liver-to-body weight ratio in Control and 3-oxo-LCA-treated offspring at 8 weeks. **I** Gene expression of IL-17, IL-17RA, and IL-17RD in liver tissues from Control and 3-oxo-LCA-treated offspring (*n* = 6 per group). **J** Gene expression of fatty acid (FA) transport, synthesis, and oxidation pathways in liver tissues from Control and 3-oxo-LCA-treated offspring (*n* = 6 per group). **K** Hepatic triglyceride (TG) content in rats from Control and 3-oxo-LCA-treated offspring (*n* = 8 per group). **L**, **M** Representative images of H&E staining and Oil Red O staining of liver tissues in rats (200× magnification). **N** Quantification of hepatic steatosis area by H&E staining in rats (*n* = 5 per group). **O** Quantification of lipid deposition by Oil Red O-positive area in rats (*n* = 5 per group). Data are presented as mean ± SEM and compared by Student’s *t*-test.

To identify the specific microbial metabolites responsible for the protective effects of lithocholic acid (LCA), we performed targeted bile acid metabolomic profiling of plasma from 3-week-old RT and Cold offspring. This analysis revealed distinct bile acid signatures between groups (Fig. 5A), with two LCA derivatives—3-oxo-LCA and isoalloLCA—significantly elevated in Cold offspring compared with RT controls (Fig. 5B). LCA is known to undergo sequential microbial transformation via 3α-hydroxysteroid dehydrogenase (3α-HSDH) to generate 3-oxo-LCA and isoalloLCA, respectively (Fig. 5C). Notably, 3-oxo-LCA has been reported to inhibit Th17 cell differentiation by antagonizing the nuclear receptor RORγt^34,42,43^. Consistent with elevated metabolite levels, Cold offspring exhibited significantly higher gut microbial abundance of 3α-HSDH-encoding genes than RT controls (Fig. 5D).

To determine whether 3-oxo-LCA is functionally sufficient to confer metabolic protection, we administered it to RT offspring maintained on a Western diet (Fig. 5E). 3-oxo-LCA supplementation markedly improved glucose tolerance and insulin sensitivity (Fig. 5F, G) and reduced the liver-to-body weight ratio, liver weight, and peritesticular fat weight (Fig. 5H and Supplementary Table1). At the molecular level, 3-oxo-LCA treatment suppressed hepatic expression of IL-17A and its receptors IL-17RA and IL-17RD (Fig. 5I), consistent with attenuated Th17 signaling. Concurrently, hepatic expression of genes governing fatty acid uptake (CD36, FABP1), lipogenesis (SREBP1c, FAS), and β-oxidation (CPT1α, PPARα) was significantly downregulated (Fig. 5J), accompanied by marked reductions in hepatic steatosis, lipid droplet accumulation, and decreased hepatic TG levels (Fig. 5K–O). To further investigate whether the effect of 3-oxo-LCA was dependent on gut microbiota, we administered 3-oxo-LCA to RT offspring together with antibiotic treatment (3-oxo-LCA+Abx). However, the metabolic benefits observed with 3-oxo-LCA supplementation were not significantly altered in the presence of antibiotics (Supplementary Fig. 8A–I). Together, these findings indicate that 3-oxo-LCA is a microbial metabolite contributing to the metabolic benefits of maternal cold exposure in offspring.

### *Clostridium scindens* supplementation elevates 3-oxo-LCA levels and alleviates Western diet-induced metabolic dysfunction

**Fig. 6:**
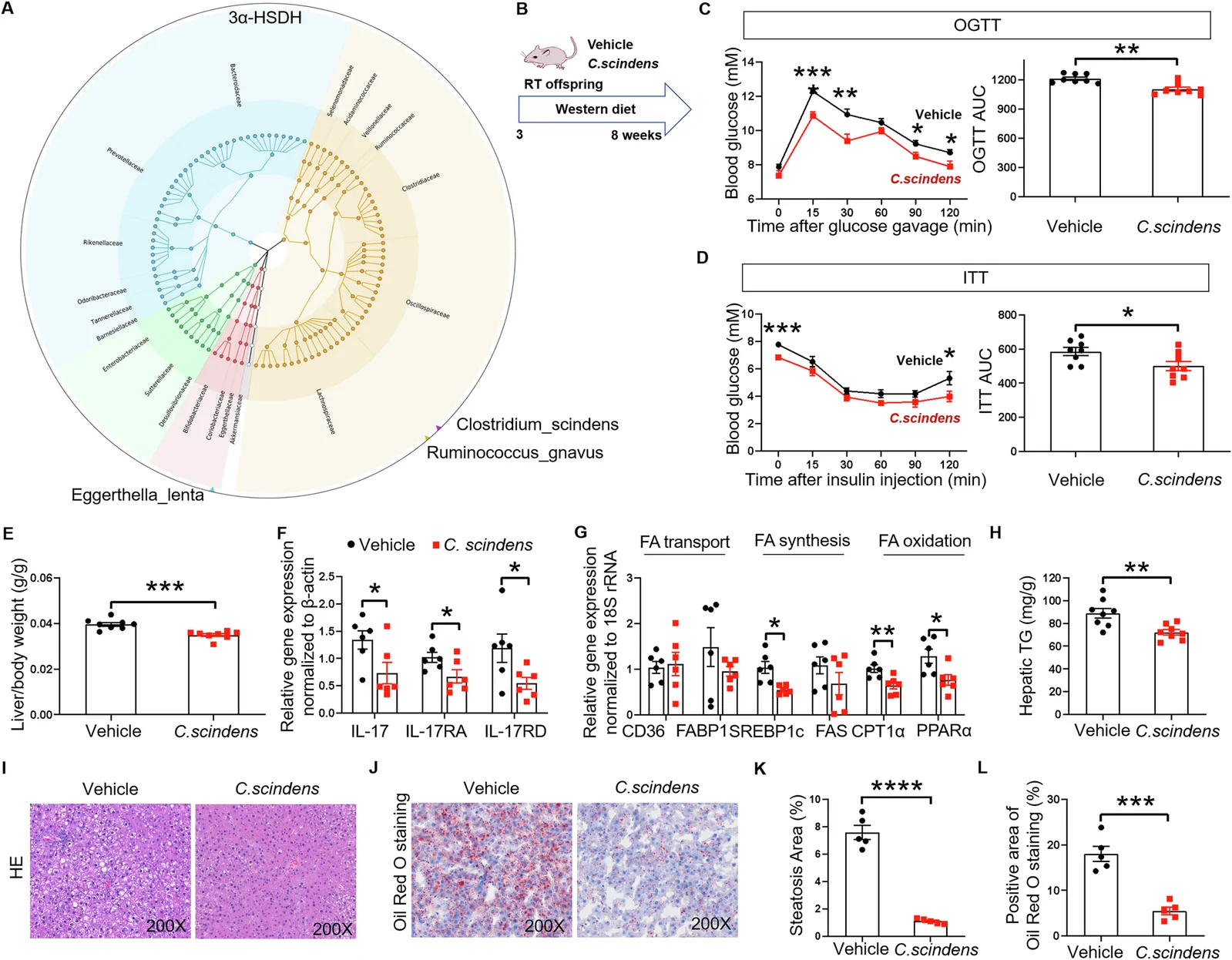
*Clostridium scindens* improves Western diet-induced glucose dysregulation and hepatic steatosis in offspring rats. **A** Distribution of the 3α-HSDH gene in 1520 bacterial reference genomes. **B** Schematic diagram of the experimental design: RT offspring were randomly assigned to Control and *C. scindens* groups. Rats received either 1 × 10⁹ CFU of *C. scindens* or vehicle by oral gavage three times per week for 5 weeks. Both groups were fed a Western diet during the intervention. **C** Oral Glucose Tolerance Test (OGTT) in vehicle- and *C. scindens*-treated offspring, showing blood glucose levels over time and the corresponding area under the curve (AUC) (*n* = 8 per group). **D** Insulin Tolerance Test (ITT) in vehicle- and *C. scindens*-treated offspring, showing blood glucose levels over time and the AUC (*n* = 8 per group). **E** Liver-to-body weight ratio in vehicle- and *C.* *scindens-*treated offspring (*n* = 8 per group). **F** The relative gene expression analysis of IL-17, IL-17RA, and IL-17RD in vehicle- and *C. scindens-* treated offspring (*n* = 6 per group). **G** The relative gene expression of fatty acid (FA) transport, synthesis, and oxidation in Vehicle- and *C. scindens-*treated offspring (*n* = 6 per group). **H** Hepatic triglyceride (TG) content in rats from the Vehicle group and the *C. scindens* group (*n* = 8 per group). **I** Representative images for H&E staining of liver tissues in rats (200× magnification. **J** Representative images for Oil Red O staining of liver tissues in rats (200× magnification). **K** Quantification of hepatic steatosis area by H&E staining in rats (*n* = 5 per group). **L** Quantification of lipid deposition by Oil Red O-positive area in rats (*n* = 5 per group). Data are presented as mean ± SEM and compared by Student’s *t*-test.

To identify key gut microbial species responsible for 3-oxo-LCA production, we systematically profiled 3α-hydroxysteroid dehydrogenase (3α-HSDH) genes across 1520 representative genomes from the human gut microbiome. This analysis revealed that 3α-HSDH-encoding genes were largely restricted to a limited subset of bacterial taxa, with *Clostridium scindens*, *Ruminococcus gnavus*, and *Eggerthella lenta* emerging as dominant contributors (Fig. 6A). To functionally validate the role of *C. scindens*, we administered this strain to RT offspring maintained on a Western diet, while control animals received vehicle alone (Fig. 6B). We confirmed successful colonization of *C. scindens* through PCR, and supplementation with *C. scindens* significantly increased the fecal levels of 3-oxo-LCA (Fig. S9A, B). Remarkably, *C. scindens* supplementation significantly improved glucose tolerance and insulin sensitivity compared with controls (Fig. 6C, D). In addition, treated offspring exhibited a lower liver-to-body weight ratio, liver weight, and peritesticular fat weight (Fig. 6E and Supplementary Table 1) and reduced hepatic expression of IL-17A, IL-17RA, and IL-17RD (Fig. 6F). Hepatic expression of key genes involved in fatty acid synthesis and β-oxidation was also markedly suppressed (Fig. 6G), in parallel with a notable reduction in hepatic triglyceride levels, hepatic steatosis, and lipid droplet accumulation (Fig. 6H–L). Collectively, these findings support *C. scindens* as a key microbial driver of 3-oxo-LCA–mediated metabolic protection in the context of Western diet-induced dysfunction.

### Association between winter conception and offspring MASLD risk in humans

**Fig. 7:**
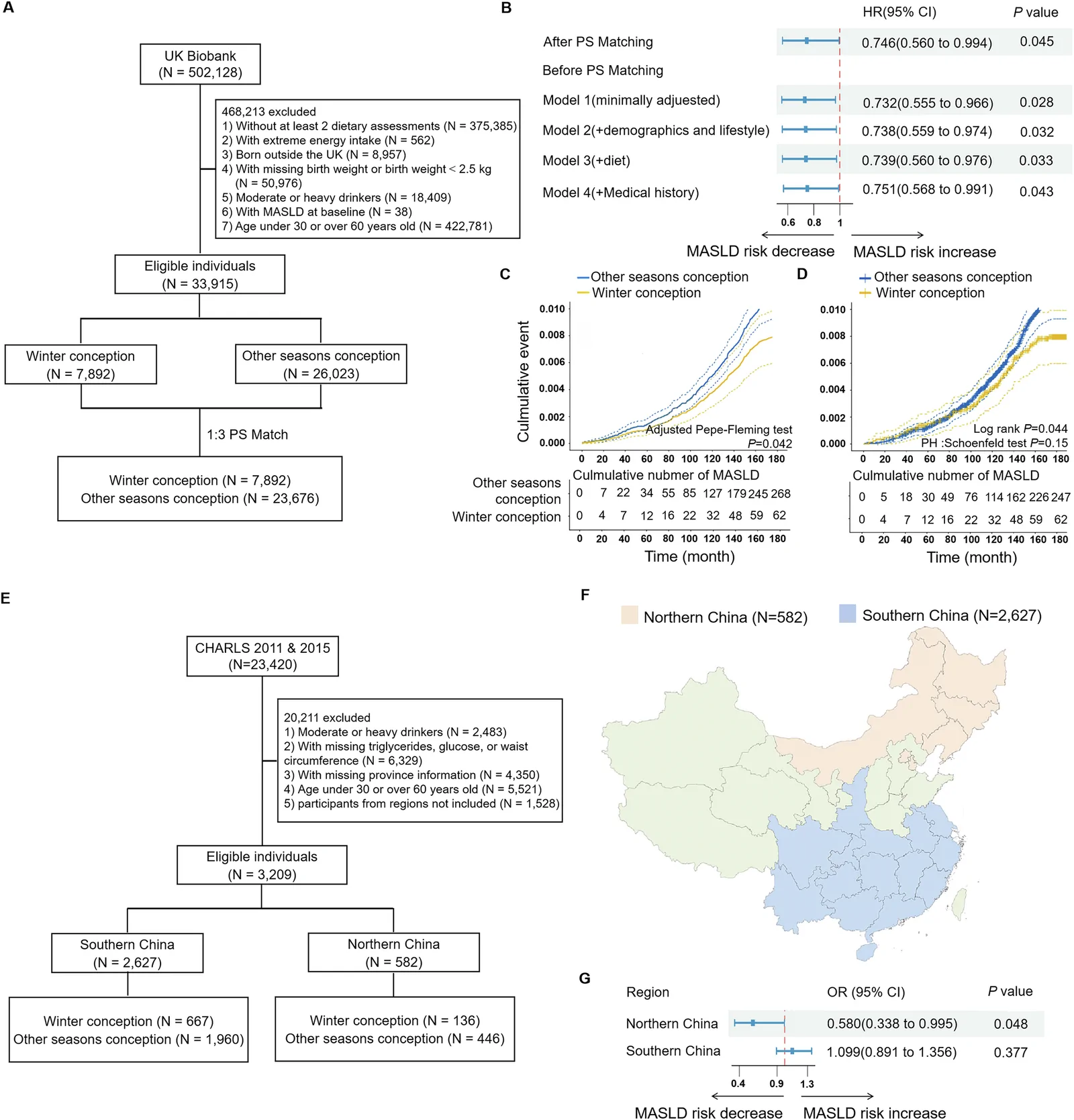
Population-based analyses of the association between winter conception and risk of MASLD in offspring. **A** Flowchart of participant selection from the UK Biobank cohort, showing inclusion and exclusion criteria and propensity score (PS) matching. **B** Forest plot depicting hazard ratios (HR) and 95% confidence intervals (CI) for MASLD risk associated with winter conception under different adjustment models and after PS matching. Models were adjusted for age, sex, race, BMI, smoking status, physical activity, and other baseline characteristics as described. Cumulative incidence curves of MASLD in offspring conceived in winter versus other seasons of conception, analyzed using the G-method and adjusted Pepe-Fleming test (**C**) and log-rank test (**D**). **E** Flow diagram illustrating the selection of participants in the CHARLS cohort. A total of 23,420 participants were initially included. After applying exclusion criteria, 20,211 individuals were excluded, resulting in a final analytic cohort of 3209 participants. **F** Geographic distribution of CHARLS participants stratified into northern and southern regions of China. **G** Odds ratios (OR) and 95% CI for MASLD risk in winter versus other seasons conception in northern and southern China, analyzed using logistic regression models adjusted for gender, age, smoking status, education level, and baseline hypertension.

To investigate whether early-pregnancy cold exposure was associated with long-term MASLD risk in offspring, we analyzed two independent population-based cohorts using winter conception as a proxy for cold exposure during early pregnancy. In the UK Biobank, 33,915 eligible participants were identified, including 7892 (23.3%) in the winter conception group and 26,023 in the other seasons conception group (Fig. 7A). In the unmatched cohort, winter conception was associated with a 24.9% lower hazard of MASLD in the fully adjusted model (Model 4; HR = 0.751; 95% CI, 0.568–0.991), this association remained consistent after progressive adjustment for potential confounders (Models 1–3) and after 1:3 propensity score matching (HR = 0.746; 95% CI, 0.560–0.994; Fig. 7B). G-method-based counterfactual analysis further supported a lower cumulative risk of MASLD in the winter conception group (Fig. 7C). Kaplan–Meier estimates in the matched cohort likewise demonstrated a lower cumulative incidence of MASLD in the winter conception group (Fig. 7D).

Furthermore, we analyzed the CHARLS cohort stratified by region, given the substantial temperature disparity between Northern (cold) and Southern (warm) China. We found that winter conception was associated with lower odds of offspring MASLD in Northern China (OR = 0.580; 95% CI, 0.338–0.995), whereas no such association was observed in the warmer South (OR = 1.099; 95% CI, 0.891–1.356) (Fig. 7E–G). Together, these results indicate that winter conception, used as a proxy for early-pregnancy cold exposure, is associated with a lower risk of MASLD in offspring.

## Discussion

**Fig. 8:**
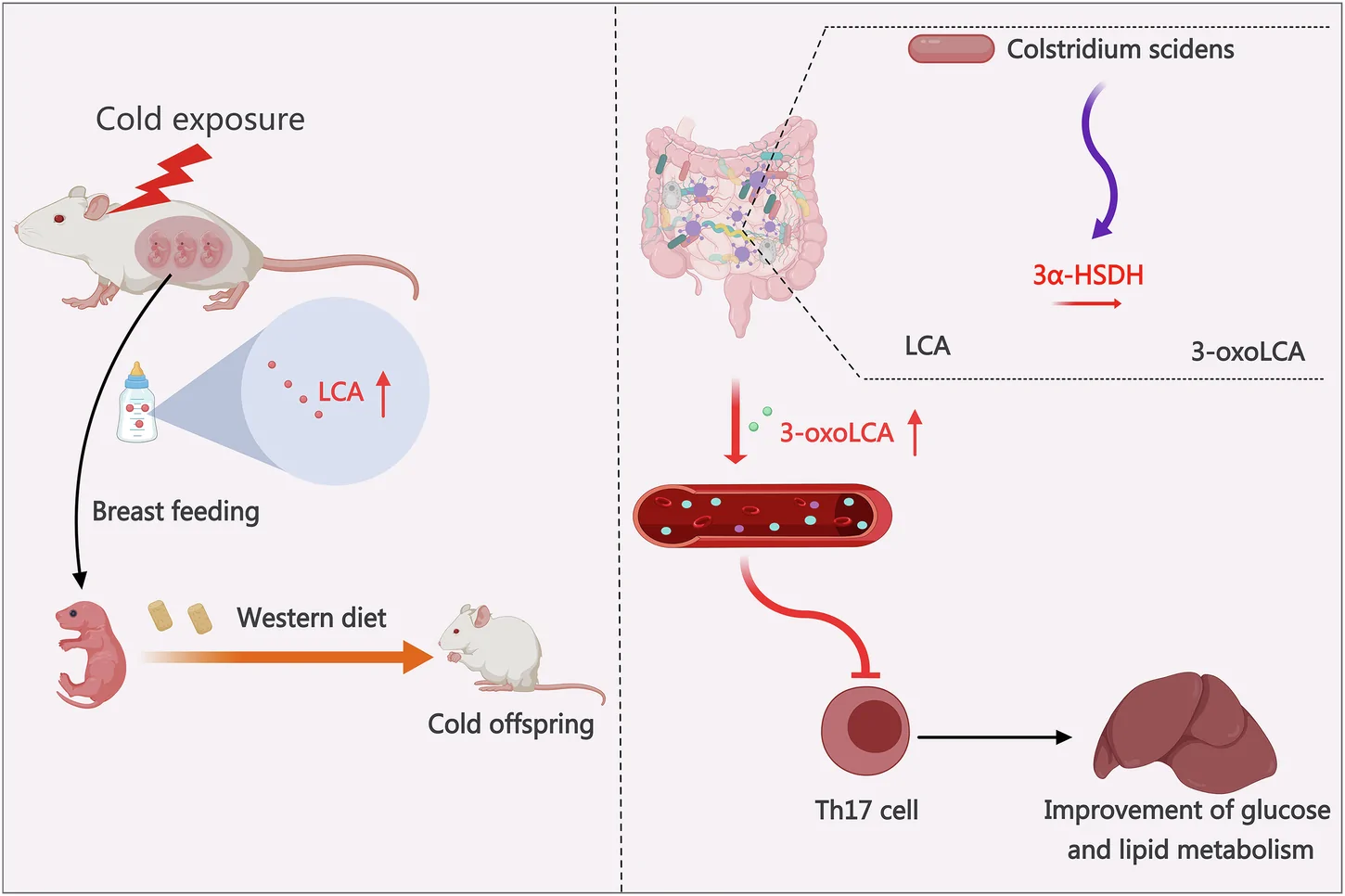
Maternal cold exposure reprograms offspring metabolism via a microbiota–bile acid–Th17 axis. Maternal cold exposure during gestation increases lithocholic acid (LCA) levels in breast milk. In the neonatal gut, LCA is converted into 3-oxo-LCA by commensal bacteria with 3α-hydroxysteroid dehydrogenase (3α-HSDH) activity, such as *Clostridium scindens*. 3-oxo-LCA suppresses Th17 cell differentiation and IL-17 signaling, improving glucose tolerance and hepatic lipid metabolism in offspring challenged with a Western diet. Together, these findings reveal a maternal cold–microbiota–bile acid–Th17 axis that contributes to long-term metabolic resilience in offspring.

Early-life environmental exposures exert profound and enduring effects on offspring metabolic health. In this study, we identified maternal cold exposure during pregnancy as a previously underexplored environmental factor that may confer long-term protection against Western diet-induced hepatic steatosis and glucose intolerance in offspring. Mechanistically, we uncover a milk–bile acid–immune axis whereby maternal cold exposure elevates LCA levels in maternal milk, and LCA is subsequently metabolized by the offspring gut microbiota into 3-oxo-LCA. This microbial metabolite suppresses proinflammatory Th17 responses in the offspring, thereby attenuating hepatic IL-17 signaling and associated disturbances in fatty acid metabolism. Our findings expand the repertoire of maternal environmental factors with durable metabolic consequences and highlight that bile acid–mediated immune modulation may be a central mechanism linking early-life exposures to adult metabolic outcomes (Fig. 8). Importantly, population-based analyses in the UK Biobank (UKB) and CHARLS cohorts further supported an inverse association between winter conception, used as a proxy for early gestational cold exposure, and future MASLD risk in offspring.

While prior studies have demonstrated that adult cold exposure activates thermogenesis and modulates systemic metabolism via brown adipose tissue^26,44–46^, our work reveals that gestational cold exposure confers intergenerational metabolic benefits without evidence of direct thermogenic activation in the offspring. The improvements in glucose tolerance and insulin sensitivity, together with reduced hepatic lipid accumulation, implicate a developmental programming mechanism that extends beyond conventional metabolic pathways. Consistent with this, recent human studies have reported that children conceived during winter exhibit enhanced adaptive thermogenesis, higher daily energy expenditure, and a lower risk of obesity^47,48^, further supporting the notion that maternal cold exposure may impart lasting metabolic advantages to the next generation. Our analysis of data from the UK Biobank cohort aligns with these findings, revealing that winter conception was associated with a lower subsequent risk of MASLD in offspring. Similarly, analysis of the CHARLS cohort revealed a similar association between winter conception and reduced MASLD risk in northern China, where winter temperatures are lower than in southern China. These population-based findings support a potential link between winter conception, used as a proxy for early gestational cold exposure, and reduced risk of MASLD in offspring. However, “winter conception” in our study was inferred by back-calculating 10 months from the participant’s birth month, representing a proxy rather than a directly measured prenatal exposure and potentially introducing exposure misclassification, including variation in gestational timing. In addition, detailed information on maternal characteristics during pregnancy, such as parental socioeconomic status at conception, pregnancy planning, maternal health, and parity, was not available in these cohorts for adjustment. As previously reported, season-of-birth associations with later-life outcomes may be partly confounded by seasonal variation in maternal characteristics and family background^49^. Therefore, residual confounding cannot be completely ruled out, and our epidemiological findings should be interpreted as associative, serving to complement the evidence derived from our animal models.

Breast milk serves as a critical conduit between maternal and infant health. Adverse maternal exposures can alter milk composition with long-term consequences for offspring health. For example, prenatal antibiotic exposure reduces breast milk IgA levels, increasing neonatal susceptibility to bacterial sepsis^50^. Maternal nanoplastic exposure has also been shown to alter breast milk lipid profiles and disrupt offspring gut microbiota, thereby promoting weight gain in progeny^51^. Conversely, beneficial maternal interventions during pregnancy can improve offspring metabolic health by modulating milk composition. Maternal exercise before and during pregnancy elevates levels of the oligosaccharide 3′-sialyllactose in breast milk, mitigating the detrimental effects of a high-fat diet on offspring metabolic health^21^. Similarly, higher maternal betaine intake during lactation increases breast milk betaine levels, improving long-term glucose homeostasis in mouse offspring^52^. In our study, cross-fostering experiments revealed that the protective effects of maternal cold exposure are mediated, at least in part, through breast milk. Specifically, we demonstrated that cold exposure during pregnancy increases LCA levels in breast milk, supporting LCA as a key candidate molecule that modulates offspring metabolic health. Although our findings support a bile acid–dependent mechanism linking maternal cold exposure to offspring metabolic protection, we cannot fully isolate the specific contribution of milk-derived LCA from other maternal or milk-associated factors. Future studies using strategies that selectively manipulate LCA levels in breast milk, without altering other maternal components, will be required to more definitively establish the role of milk-derived LCA.

Bile acids are critical metabolic regulators that facilitate dietary fat digestion and absorption while also modulating whole-body metabolism^30^. Alterations in bile acid synthesis and composition have been implicated in the development of obesity, insulin resistance, and other metabolic disorders^27,29^. Primary bile acids are synthesized by hepatocytes and converted by intestinal bacteria into secondary bile acids^53^. Breast milk contains bile acids, and their levels have been shown to influence neonatal susceptibility to viral infections in murine models^54^.

LCA, one of the predominant secondary bile acids, has been reported to activate AMP-activated protein kinase, mimicking the anti-aging effects of caloric restriction^33^. In our study, we found that maternal cold exposure increased LCA levels in breast milk, which is further metabolized by the offspring gut microbiota into 3-oxo-LCA and isoalloLCA. We identify 3-oxo-LCA as a functionally relevant metabolite that directly contributes to metabolic improvements. Prior studies have shown that 3-oxo-LCA inhibits Th17 cell differentiation^34^, a pathway closely linked to hepatic metabolic disease^55,56^. Consistent with this, we showed that maternal cold exposure was associated with reduced circulating Th17 cell populations and hepatic IL-17A expression in offspring. Moreover, direct 3-oxo-LCA supplementation lowers hepatic IL-17A levels and improves fatty acid metabolism in the liver. Together, our results delineate a novel bile acid–Th17 immune axis that links maternal environmental exposures to offspring metabolic health. This axis is modifiable at multiple levels—including maternal bile acid composition, neonatal gut microbial colonization, and microbial bile acid biotransformation—offering multiple potential points for therapeutic intervention. Notably, our findings suggest that 3-oxo-LCA or its microbial producers, such as *C. scindens*, could serve as tractable targets for microbiota- or bile acid–based therapies aimed at mitigating diet-induced metabolic dysfunction. However, a potential limitation of this study is that in vitro mechanistic experiments were performed using human cell lines (HepG2 and Jurkat), whereas bile acid metabolites were identified in rat breast milk. Given known species-specific differences in bile acid metabolism and receptor signaling, the cellular responses observed in human cells may not fully recapitulate those occurring in rat tissues. Future studies employing primary rat hepatocytes or immune cells will be required to more precisely define species-specific mechanisms.

Several limitations should be acknowledged. First, although we demonstrate the functional relevance of 3-oxo-LCA in rat models, its translatability to human neonates remains to be established. Second, in the human cohort analyses, bile acids, including LCA, were not measured; therefore, the human data provide supportive phenotypic evidence but do not directly establish the proposed LCA–metabolic mechanism. In addition, the lack of individual-level data on outdoor duration in the UK Biobank limited our ability to precisely quantify personal cold exposure, introducing uncertainty in exposure assessment. Finally, we were unable to precisely determine the absorption kinetics of exogenously supplemented LCA or 3-oxo-LCA, which may influence the interpretation of their systemic effects.

Overall, our study identifies maternal cold exposure as a previously underexplored environmental factor that contributes to durable changes in offspring metabolic health. By revealing a modifiable link between maternal milk–bile acid profiles, offspring gut microbiota, and immune signaling, our findings provide a mechanistic framework for intergenerational modulation of metabolic outcomes. These results not only deepen our understanding of how early-life environmental exposures shape long-term metabolic trajectories but also support future clinical investigations aimed at optimizing maternal interventions during pregnancy to reduce offspring risk of metabolic disorders.

## Methods

### UK Biobank cohort

Data were obtained from the UK Biobank (UKB), a population-based cohort of ~500,000 participants aged 37–73 years from the UK. The UK Biobank study received ethics approval from the North West-Haydock Research Ethics Committee (reference 11:/NW/0382), ensuring the protection of participants and adherence to the principles of the Declaration of Helsinki. All participants provided written informed consent at enrolment. For this analysis, we included 30–60-year-old participants who completed at least two 24-h online dietary questionnaires, were born in the United Kingdom, and had a birth weight ≥2.5 kg. Participants with implausible energy intake, moderate or heavy alcohol consumption, or prevalent MASLD at baseline were excluded. Prevalent MASLD was defined as the presence of ICD-10 code K76.0 recorded in linked hospital inpatient records prior to the baseline assessment date in UK Biobank. The current analysis was performed under UK Biobank application number 532351.

Winter conception was defined by subtracting 10 months from each participant’s birth month; December–February were classified as winter, all other months as non-winter. MASLD was defined according to current diagnostic criteria (metabolic dysfunction-associated steatotic liver disease), excluding participants with alcohol intake above recommended thresholds.

### CHARLS cohort

CHARLS is a nationally representative cohort of Chinese adults aged ≥45 years. The CHARLS data collection was approved by the Biomedical Ethics Review Committee of Peking University (IRB00001052-11015). All participants provided written informed consent. We used cross-sectional data from the CHARLS 2011 baseline survey and the CHARLS 2015 follow-up survey. We included participants aged 30–60 years who had data on triglycerides, fasting glucose, and waist circumference. Individuals with viral hepatitis or excessive alcohol consumption were excluded from the analysis. In the CHARLS cohort, direct assessments of hepatic steatosis, including liver imaging or histological evaluation, were not available. Therefore, MASLD was operationally defined using the TyG-WC index, which integrates fasting triglycerides, fasting glucose, and waist circumference. This index has been widely used as a validated surrogate indicator for fatty liver disease in epidemiological studies where direct assessment of liver condition is not available^57,58^.

### Statistical analysis of cohorts

Associations between winter conception and MASLD were estimated using Cox proportional hazards regression in UKB and logistic regression in CHARLS, adjusting for relevant covariates, including age, sex, smoking, alcohol intake, and socio-demographic factors. Significance was set at *P* < 0.05. Analyses were performed in R 4.5.1 (R Foundation, Vienna, Austria).

### Experimental animals

The animal experimental protocol adhered to the Directive 2010/63/EU of the European Parliament on animal welfare in research and the current standards outlined by the U.S. National Institutes of Health, and the study was designed and reported in accordance with the ARRIVE 2.0 guidelines for animal experiments. 8-week-old Sprague-Dawley rats were purchased from Beijing Vital River Laboratory Animal Technology Co. Ltd (Beijing, China) and housed in SPF conditions with light/dark cycles of 12 h and had access to food and water ad libitum. After acclimation, female and male rats were mated at a ratio of 2:1. After cohabitation, vaginal spermatozoa were examined daily to confirm pregnancy, and if found, the day was denoted as gestation day 0.5. After pregnancy, female rats were randomly exposed to room temperature (RT) (25 ± 1 °C) or cold exposure (4 ± 1 °C) in a dedicated temperature-controlled ventilated chamber (HWS-500, Jiangsu Tianling Instrument Co., Ltd., China) during the first 10 days of gestation. Their offspring were respectively referred to as RT offspring and Cold offspring, and all were raised under RT conditions after birth. To address potential litter-specific confounding, each experimental group included offspring derived from different dams, with two offspring selected from the same litter. During the pre-weaning period, offspring remained with their dams and were allowed to breastfeed ad libitum. To standardize maternal care and nutritional exposure, each dam nursed 8–10 pups. At weaning (postnatal 21 days), pups were separated from dams and placed in cages with access to food and water ad libitum until 8 weeks old. No animals were excluded after allocation. Selection was not based on body weight or metabolic outcomes. Data regarding the terminal body weight, liver weight, and perigonadal fat weight across all experimental animals can be found in Supplementary Table 1.

In our study, both male and female offspring were initially analyzed. Significant metabolic differences between the room-temperature and cold-exposed groups were observed only in male offspring, with no comparable changes in females. Therefore, to avoid potential confounding by sex, all subsequent analyses were conducted using male offspring only.

At the end of the experiments, all rats were anesthetized by intraperitoneal injection of an overdose of sodium pentobarbital (150 mg/kg) and euthanized via cervical dislocation, followed by exsanguination to confirm death. Tissue samples were collected immediately for further analysis.

### In vivo randomization and blinding procedures

Randomization and blinding procedures were implemented to ensure impartiality. All animals were labeled with unique identifiers according to the animal facility’s labeling system and were randomly allocated to experimental groups to minimize selection bias. For downstream experiments requiring a subset of animals (e.g., hepatic RNA-seq), animals were randomly selected from each group only after the completion of all in vivo metabolic measurements. The investigator performing the selection and all subsequent analyses was blinded to the group allocation and individual metabolic phenotypes.

### Western diet regimen

Following weaning, offspring were maintained on a Western diet regimen consisting of a high-fat diet containing 60% kcal from fat, 16.1% kcal from protein, and 23.9% kcal from carbohydrates (MD12033, Medicience Ltd.), together with 10% (w/v) fructose in the drinking water.

### Supplementation of LCA and antibiotic intervention

For LCA supplementation, 3-week-old RT offspring were randomly divided into a vehicle control group, LCA group, and LCA+Abx group. Rats in the LCA group received 100 μL LCA by oral gavage (HY-B0172, MCE, 50 mg/kg/day in corn oil) starting at 3 weeks of age for 5 consecutive weeks. Rats in the LCA+Abx group received LCA by oral gavage, together with continuous exposure to antibiotics administered via drinking water. The antibiotic cocktail consisted of 0.5 g/L vancomycin, 1 g/L ampicillin, 1 g/L neomycin, and 1 g/L metronidazole, and was provided ad libitum from 3 weeks of age for 5 consecutive weeks. The antibiotic-containing water was refreshed regularly to ensure consistent exposure. All groups were fed a Western diet from 3 weeks of age for 5 weeks.

### Supplementation of 3-oxo-LCA

For supplementation of 3-oxo-LCA, RT offspring were randomly divided into a control group and a 3-oxo-LCA group, and all of them received a Western diet. Rats in the 3-oxo-LCA group received 100 μL of 3-oxo-LCA (O351925, Aladdin, 50 mg/kg/day in corn oil) by gavage from 3 weeks of age for 5 weeks. Rats in the vehicle group received daily gavage of an equal volume of corn oil. Both groups were fed a Western diet from 3 weeks of age for 5 weeks.

### Supplementation of 3-oxo-LCA and antibiotic intervention

3-week-old RT offspring were randomly assigned to either the 3-oxo-LCA group or the 3-oxo-LCA+Abx group. Rats in both groups received 100 μL of 3-oxo-LCA by oral gavage (50 mg/kg/day) from 3 weeks of age for 5 consecutive weeks. Rats in the 3-oxo-LCA+Abx group additionally received drinking water containing an antibiotic cocktail composed of 0.5 g/L vancomycin, 1 g/L ampicillin, 1 g/L neomycin, and 1 g/L metronidazole. The antibiotic-containing water was provided ad libitum for 5 consecutive weeks. Both groups were fed a Western diet from 3 weeks of age for 5 weeks.

### Cross-fostering

Within 24 h after birth, all rat litters were cross-fostered to clearly distinguish the effects of gestational exposure (biological origin) from the postnatal rearing environment (foster dam). The group names are explicitly defined by the foster dam condition, followed by the biological origin of the offspring. Specifically, litters born to cold-exposed mothers were fostered and nursed by room-temperature (RT) mothers (RT + Co group, indicating RT foster dams + Cold-origin offspring). Conversely, litters born to RT mothers were fostered and nursed by cold-exposed mothers (Cold+Ro group, indicating Cold foster dams + RT-origin offspring). Litter pups were fed by their respective foster mothers until weaning at 3 weeks of age, after which all offspring were fed a Western diet for 5 weeks.

### Cultivation and oral administration of *Clostridium scindens*

*Clostridium scindens* (BNCC341871) was purchased from BeNa Culture Collection (BNCC, Beijing, China). It was routinely cultured on Columbia Blood Agar (BNCC352241) at 37 °C in an anaerobic chamber using a gas mix consisting of 10% hydrogen, 10% carbon dioxide, and 80% nitrogen for 48–72 h. For *Clostridium scindens* supplementation, bacteria were collected by centrifugation at 3500 rpm/min for 10 min and resuspended in sterilized saline with 20% (v/v) glycerol. Rats were treated with 1 × 10^9^ colony-forming units (CFU) of *Clostridium scindens* by gavage, three times a week for 5 weeks.

### Isolation of breast milk

SD pregnant rats were randomly divided into two groups, and exposed to RT (25 ± 1 °C) or Cold (4 ± 1 °C) from G0.5 to G10.5. Milk was collected 7 days after birth. Pups were separated from the dams 24 h before milk collection. Using an established method^21^, mammary glands were carefully excised and immediately placed on a sterile dish on ice. To reduce potential contamination from tissue-derived fluids or blood, only milk passively released onto the dish over 4 h was collected, without any mechanical squeezing or tissue disruption. The collected milk was then centrifuged at 1500 × *g* for 15 min at 4 °C to remove residual debris, and the supernatant was stored at −80 °C until further analysis.

### LC-MS/MS analysis

LC-MS/MS analysis was performed on a Vanquish UHPLC system (Thermo Fisher Scientific) equipped with a UPLC BEH Amide column (2.1 mm × 100 mm, 1.7μm), coupled to a Q Exactive HFX Orbitrap mass spectrometer (Thermo Fisher Scientific). The mobile phases consisted of 25 mmol/L ammonium acetate and 25 mmol/L ammonium hydroxide in water (pH 9.75) as phase A, and acetonitrile as phase B. The column temperature was maintained at 25 °C, the autosampler at 4 °C, and the injection volume was 2 μL. Mass spectrometry data were acquired in data-dependent acquisition (DDA) mode using Xcalibur software (Thermo Fisher Scientific). The ESI source parameters were: sheath gas flow rate, 30 arbitrary units; auxiliary gas flow rate, 25 arbitrary units; capillary temperature, 350 °C; spray voltage, +3.6 kV (positive mode) and −3.2 kV (negative mode). Full MS resolution was set at 60,000, and MS/MS resolution at 7500. Normalized collision energies (NCE) were set at 10, 30, and 60.

### Bile acid analysis by UHPLC-PRM-MS

Bile acids were analyzed using ultra-high-performance liquid chromatography (UHPLC) coupled with a high-resolution mass spectrometer. UHPLC separation was performed using a Vanquish UHPLC system (Thermo Fisher Scientific), equipped with a Waters ACQUITY UPLC BEH C18 column (150 mm × 2.1 mm, 1.7 μm). The mobile phase consisted of 5 mmol/L ammonium acetate in water (A) and acetonitrile (B). The column temperature was maintained at 45 °C, and the autosampler temperature was set to 4 °C. A 1 μL injection volume was used for each sample. Mass spectrometric analysis was carried out with an Orbitrap Exploris 120 mass spectrometer (Thermo Fisher Scientific) in parallel reaction monitoring (PRM) mode. The typical ion source parameters were as follows: spray voltage = +3500/−3200 V, sheath gas flow rate = 40, auxiliary gas flow rate = 15, sweep gas flow rate = 0, auxiliary gas temperature = 350 °C, and capillary temperature = 320 °C. To avoid interference from structurally related bile acids, isomers, and modified bile acid species, bile acids were identified using monitored ion pairs together with accurate mass matching (mass tolerance <5 ppm) and chromatographic retention time matching with authentic bile acid reference standards analyzed under identical UHPLC-HRMS conditions. Chromatographic separation on the ACQUITY UPLC BEH C18 column provided sufficient resolution of structurally related bile acids prior to mass spectrometric detection.

### RNA sequencing and analysis

Total RNA was extracted and subjected to high-throughput sequencing using standard protocols by Novogene Co., Ltd. (Beijing, China). Libraries were prepared with the NEBNext Ultra RNA Library Prep Kit (NEB, USA) and sequenced on an Illumina NovaSeq 6000 platform (paired-end, 150 bp). Differential gene expression was determined based on a log₂ fold change threshold of ≥1.0 or ≤–1.0, with an adjusted *p*-value (padj) <0.05 considered statistically significant.

### Flow cytometry and intracellular cytokine assay

Spleen and blood were isolated in a sterile environment. Lymphocytes were isolated from the spleen and blood using lymphocyte separation medium (Shenzhen Dakewe Bio-engineering Co, Ltd). Lymphocytes were plated in complete medium (RPMI, 10%FBS, 80 mg/dl glucose, 10 g/L HEPES, and 10 g/L glutamine, 1% penicillin and streptomycin), and stimulated with Brefeldin A (BioLegend, USA) and PMA/ionomycin(BioLegend, USA) for 5 h at 37 °C. After incubation, cells were washed with PBS and centrifuged at 500 × *g* for 5 min, then stained with extracellular antibodies, including anti-CD3-FITC (BioLegend, USA), anti-CD8-PE-Cy7 (BioLegend, USA), for 30 min on ice in the dark. Lymphocytes were fixed with IC Fixation Buffer (Invitrogen, USA), and then intracellular antibodies were added to samples and incubated on ice in the dark, including APC anti-mouse/rat IL-17A (Invitrogen, USA), and PE anti-rat RORγt (Invitrogen, USA). Flow cytometry was performed on a BD FACSCanto II (BD Biosciences, USA) and analyzed using FlowJo (Tree Star, Ashland, Ore).

### Glucose homeostasis

For oral glucose tolerance testing (OGTT), all rats were placed in clean cages and provided with water but without food. After 8 h of fasting, the rats were administered an oral glucose (2 g/kg, Sigma-Aldrich). Blood glucose levels were measured before and after oral glucose administration at 15, 30, 60, 90, and 120 min and determined with a glucose meter (Accu-Chek Performa, Roche) on blood samples collected from the tip of the tail vein. For insulin tolerance testing (ITT), all rats were placed in clean cages and provided with water but no food. After 5 h of fasting, the rats were injected with insulin (0.75 U/kg, Sigma-Aldrich), and blood glucose levels were measured before and after injection at 15, 30, 60, 90, and 120 min. To allow for complete recovery from fasting and handling stress, the ITT was performed 7 days after the OGTT.

### Histopathology

The tissues of rats were collected in 4% paraformaldehyde and then embedded in paraffin. The sections were continuously cut into 4 μm and stained with hematoxylin and eosin (HE) (Solarbio, China) in accordance with the manufacturer’s instructions. Histopathological changes were studied through light microscopy.

### Quantitative polymerase chain reaction and quantitative reverse-transcription polymerase chain reaction

Microbial DNA from a fecal sample was extracted using the TIANamp Stool DNA Kit (cat#DP328-02, TIANGEN, Beijing, China) according to the manufacturer’s instructions. The relative abundance of 3α-HSDH in stool samples was quantified by quantitative polymerase chain reaction (qPCR). qPCR assay (SYBR Green Assay, Roche, Switzerland) was performed on an Applied Biosystems.

Total RNA was extracted from liver tissues using the AxyPrep™ Multi-Source Total RNA Miniprep Kit (Axygen, USA) according to the manufacturer’s instructions, and then total RNA was reverse-transcribed into cDNA using the Tyobo 301 kit. Quantitative real-time PCR (qRT-PCR) was performed to measure mRNA expression levels. For genes related to energy metabolism, 18S rRNA was used as the reference gene for normalization. For inflammatory markers, β-actin served as the internal control. Relative gene expression was calculated using the 2^−ΔΔCT^ method.

### Profiling 3α-HSDH-encoding genes in 1520 gut reference genomes

The protein-coding sequences of 1520 reference genomes from cultivated human gut bacteria were downloaded from NCBI. The 3α-HSDH sequences retrieved from UniRef90 were searched against the protein-coding sequences of the 1520 reference genomes using BlastP with a cut-off of identity >80%, coverage > 80%, and e-value < 1e-5.

### Cell culture

Jurkat cells (a human T-cell acute lymphoblastic leukemia cell line) were purchased from Zhejiang Meisen Cell Technology Co., Ltd. (CTCC-400-0075) and cultured in RPMI-1640 medium supplemented with 10% fetal bovine serum (FBS) and 1% penicillin-streptomycin in an incubator at 37 °C with 5% CO2. HepG2 cells (a human hepatocellular carcinoma cell line) were obtained from BNCC, China (BNCC-338070), and maintained in MEM supplemented with 10% FBS and 1% penicillin-streptomycin in an incubator at 37 °C with 5% CO_2_. When HepG2 and Jurkat cells reached approximately 60–70% confluence, they were divided into three groups: a control group, a PA group, and a PA + LCA group. Cells in the PA and PA + LCA groups were treated with 200 μM palmitic acid (PA), while the PA + LCA group received an additional 10 μM lithocholic acid (LCA). After 24 h of treatment, relevant cellular and molecular parameters were assessed.

### Statistical analysis

Statistical analysis was performed using GraphPad Prism 8.0 software (GraphPad Software, Inc, La Jolla, CA). Shapiro-Wilk test was used for normality. Continuous variables are expressed as the mean ± standard error of the mean (SEM) or median and interquartile range. Categorical variables are represented as numbers and percentages; the comparison between two groups was assessed by unpaired Student’s *t*-test or Wilcoxon (Mann–Whitney U) test. Variables with more than two groups were analyzed by one-way ANOVA followed by Tukey tests or Kruskal–Wallis followed by a post hoc test with Bonferroni adjustment. The χ^2^ test was used for the comparison of categorical variables. *P* < 0.05 indicated statistical significance between the two groups.

### Ethics approval

The animal experiments in this study were performed in accordance with the Guide for the Care and Use of Laboratory Animals and were approved by the Animal Care and Utilization Committee of Harbin Medical University (Ethical approval number: 2021144).

## Supplementary information

Supplementary information

Supplementary information

## Data Availability

The raw RNA-sequencing data generated in this study have been deposited in the NCBI Sequence Read Archive under BioProject accession number PRJNA1479571. The processed data and other data supporting the findings of this study are available within the article and its supplementary materials.
