# Supplementary material for: Maternal cold exposure improves offspring metabolic health via a milk lithocholic acid–microbiota–Th17 axis
Source: NPJ Biofilms Microbiomes. 2026 Jul 9;12:218. doi: 10.1038/s41522-026-01092-7 (PMC13724693; doi:10.1038/s41522-026-01092-7)
Supplement: Supplementary file 1 [file 41522_2026_1092_MOESM1_ESM.pdf]

Supplementary materials

Supplementary Table 1. Liver weight, body weight, and peritesticular fat weight of offspring across experimental groups.

	liver weight	body weight	Peritesticular fat weight
RT offspring	13.79±1.77	314.38±13.92	3.26±0.22
Cold offspring	11.26±1.16**	307±26.31	2.64±0.58
Ro+SD	23.31±2.03	566±54.43	5.98±0.56
Co+SD	21.95±1.89	541.13±53.42	5.15±0.62
RT+Co	13.59±1.2	325.43±17.75	3.16±0.25
Cold+Ro	12.52±0.59	322.14±19.52	2.52±0.63*
Control	13.55±1.4	322.13±9.23	3.66±0.2
LCA	9.48±1.76***	329.25±12.31	3.46±0.27
LCA+Abx	12.78±1.29$^{\#\#}$	325.25±8.71	3.78±0.42
Control	15.88±1.75	318.38±11.1	3.64±0.26
3-oxoLCA	12.51±1.28**	320.25±11.02	2.87±0.41***
Vehicle	12.55±1.03	316±13.11	3.47±0.34
C.scindens	11.06±0.96*	316.25±23.34	2.85±0.35**

Normality was assessed using the Shapiro–Wilk test. For normally distributed data, comparisons between two groups were performed using Student's t-test, and comparisons among multiple groups were performed using one-way ANOVA followed by Tukey's post hoc test. For non-normally distributed data, the Mann–Whitney U test or Kruskal-Wallis followed by a post hoc test with Bonferroni adjustment was applied as appropriate. Statistical significance was defined as $^{*}P < 0.05$, $^{**}P$ or $^{\#\#}P < 0.01$, and $^{***}P < 0.001$.

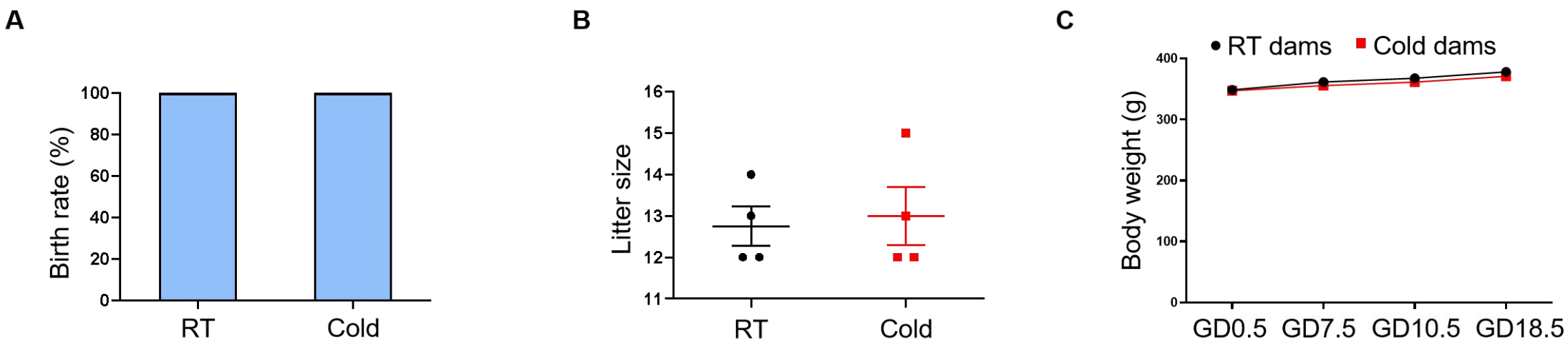

Supplementary Figure 1. Maternal cold exposure has no impact on birth rate, litter size, and maternal body weight.

(A) The birth rate of RT and Cold groups (n=4 per group).

(B) The litter size of RT and Cold groups (n=4 per group).

(C) Maternal body weight trajectories during pregnancy under room temperature (RT) or cold exposure (Cold) (n=4 per group).

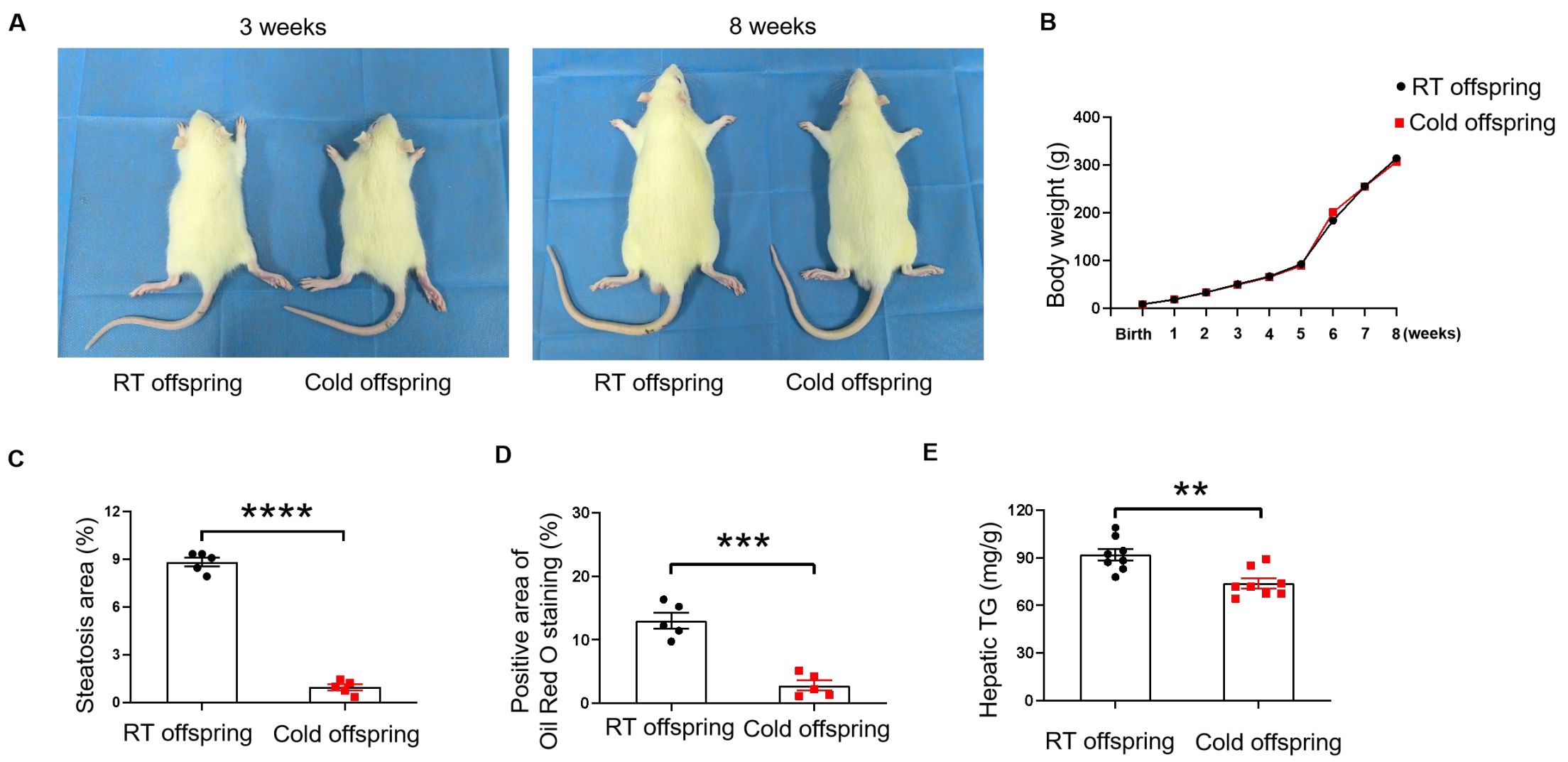

Supplementary Figure 2. Maternal cold exposure does not affect offspring body growth but reduces hepatic steatosis under Western diet challenge.

(A) Representative images of male RT and Cold offspring at 3 and 8 weeks of age.

(B) Body weight trajectories of male RT and Cold offspring from birth to 8 weeks.

(C–D) Hepatic lipid accumulation assessed by H&E staining (C) and Oil Red O staining (D) (n=5 per group).

(E) Hepatic triglyceride (TG) content in RT and Cold offspring (n=8 per group).

Data are presented as mean ± SEM. Statistical analyses were performed using Student's t-test.

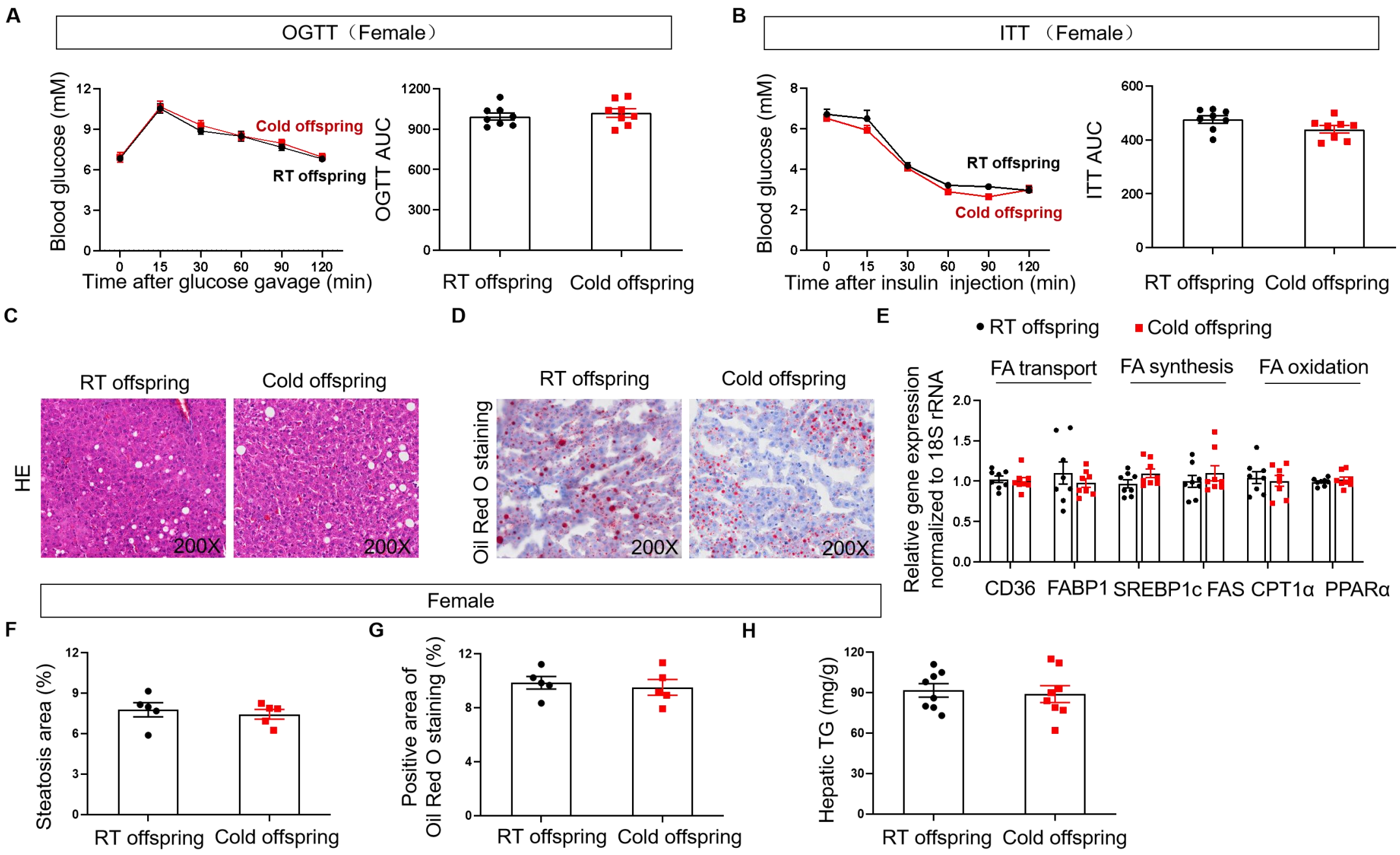

Supplementary Figure 3. Maternal cold exposure has no significant protective effect on the metabolic phenotype of female offspring.

(**A**) Oral Glucose Tolerance Test (OGTT) in 8-week-old female RT and Cold offspring, with blood glucose curves and corresponding area under the curve (AUC) (n = 8 per group).

(**B**) Insulin Tolerance Test (ITT) in 8-week-old female RT and Cold offspring, showing blood glucose levels over time and the AUC (n = 8 per group).

(**C** and **D**) Representative images for H&E and Oil Red O staining of liver tissues in female RT and female Cold offspring (n = 8 per group) (200× magnification).

(E) The relative gene expression of fatty acid (FA) transport, synthesis, and oxidation pathways in 8-week-old female RT and female Cold offspring (n = 8 per group).

(F) Quantification of hepatic steatosis area by H&E staining in rats (n=5 per group).

(G) Quantification of lipid deposition by Oil Red O-positive area in rats (n=5 per group).

(H) Hepatic triglyceride (TG) content (n=8 per group).

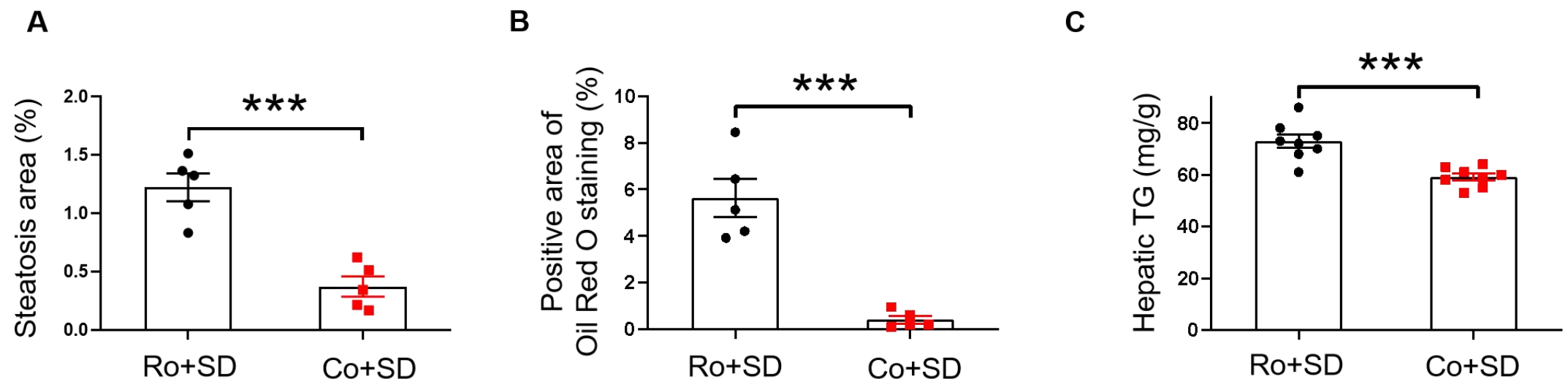

Supplementary Figure 4. Cold maternal feeding reduces hepatic steatosis and triglyceride levels in RT offspring under Western diet challenge.

(A) Quantification of hepatic steatosis area by H&E staining in rats (n=5 per group).

(B) Quantification of lipid deposition by Oil Red O-positive area in rats (n=5 per group).

(C) Hepatic triglyceride (TG) content (n=8 per group).

Data are presented as mean ± SEM and compared by Student's t test.

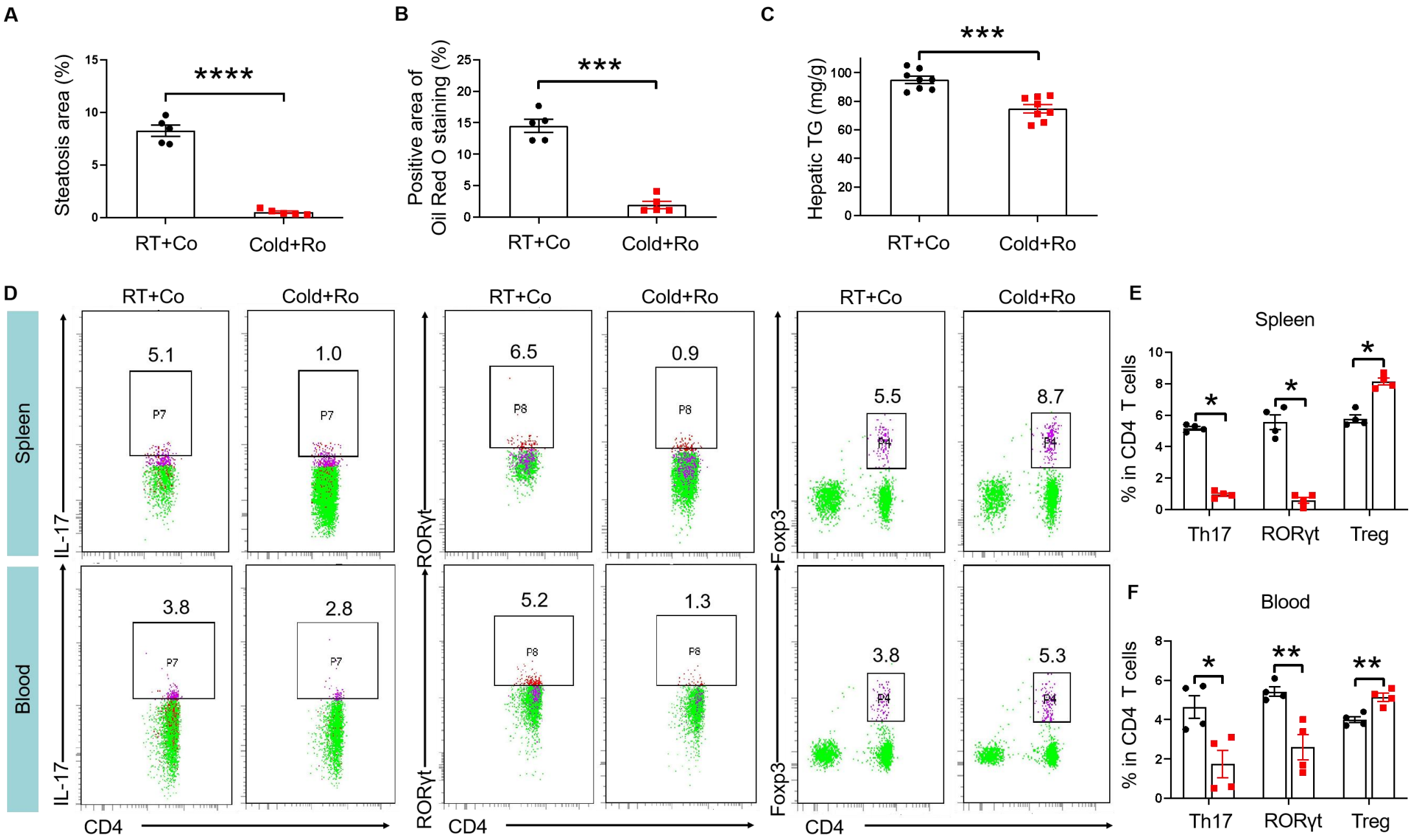

Supplementary Figure 5. Cold mother fostering reduces Th17 cell levels and hepatic steatosis in RT offspring under Western diet challenge.

(A) Quantification of hepatic steatosis area by H&E staining in rats (n=5 per group).

(B) Quantification of lipid deposition by Oil Red O-positive area in rats (n=5 per group).

(C) Hepatic triglyceride (TG) content (n=8 per group).

(D) Flow cytometry analysis of Th17, RoRγt, and Treg cells in the spleen and blood of rats.

(**E** and **F**) Quantification of the percentage of Th17, RoRγt, and Treg cells in the spleen and blood of rats (n=4 per group).

Data are presented as mean ± SEM and compared by Student's t test (A-C) or Mann–Whitney U test (E and F).

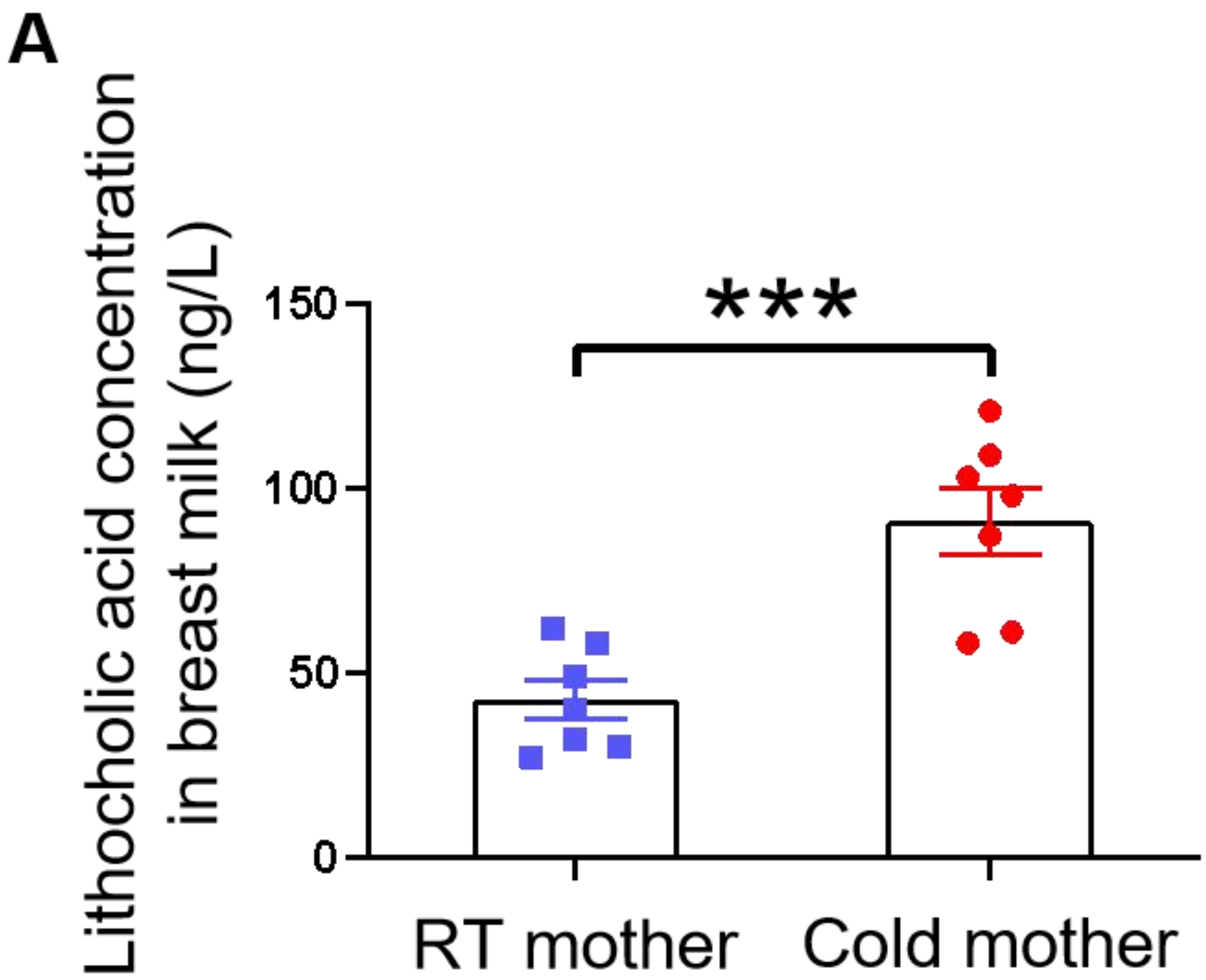

Supplementary Figure 6. The levels of lithocholic acid in the breast milk of RT mother and Cold mother.

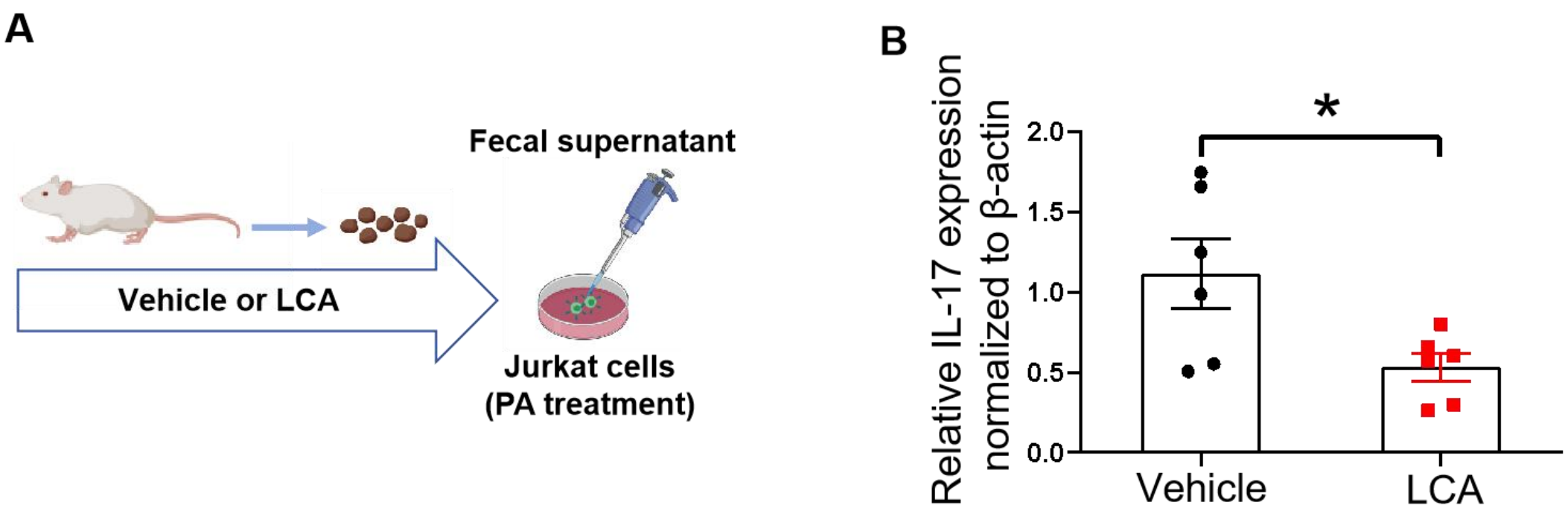

Supplementary Figure 7. Lithocholic acid promotes IL-17A secretion through its microbiota-derived metabolites.

(A) Experimental schematic: Rats were randomly divided into two groups, receiving either lithocholic acid or a control solvent for a two-week period. The dosage of lithocholic acid was 50 mg/kg/day, administered once daily. After two weeks, fecal samples were collected from both groups, and fecal microbiota supernatants were prepared. These supernatants were then used to treat Jurkat cells for 24 hours.

(B) Relative expression levels of IL-17A in the two groups of cells (n=6 per group).

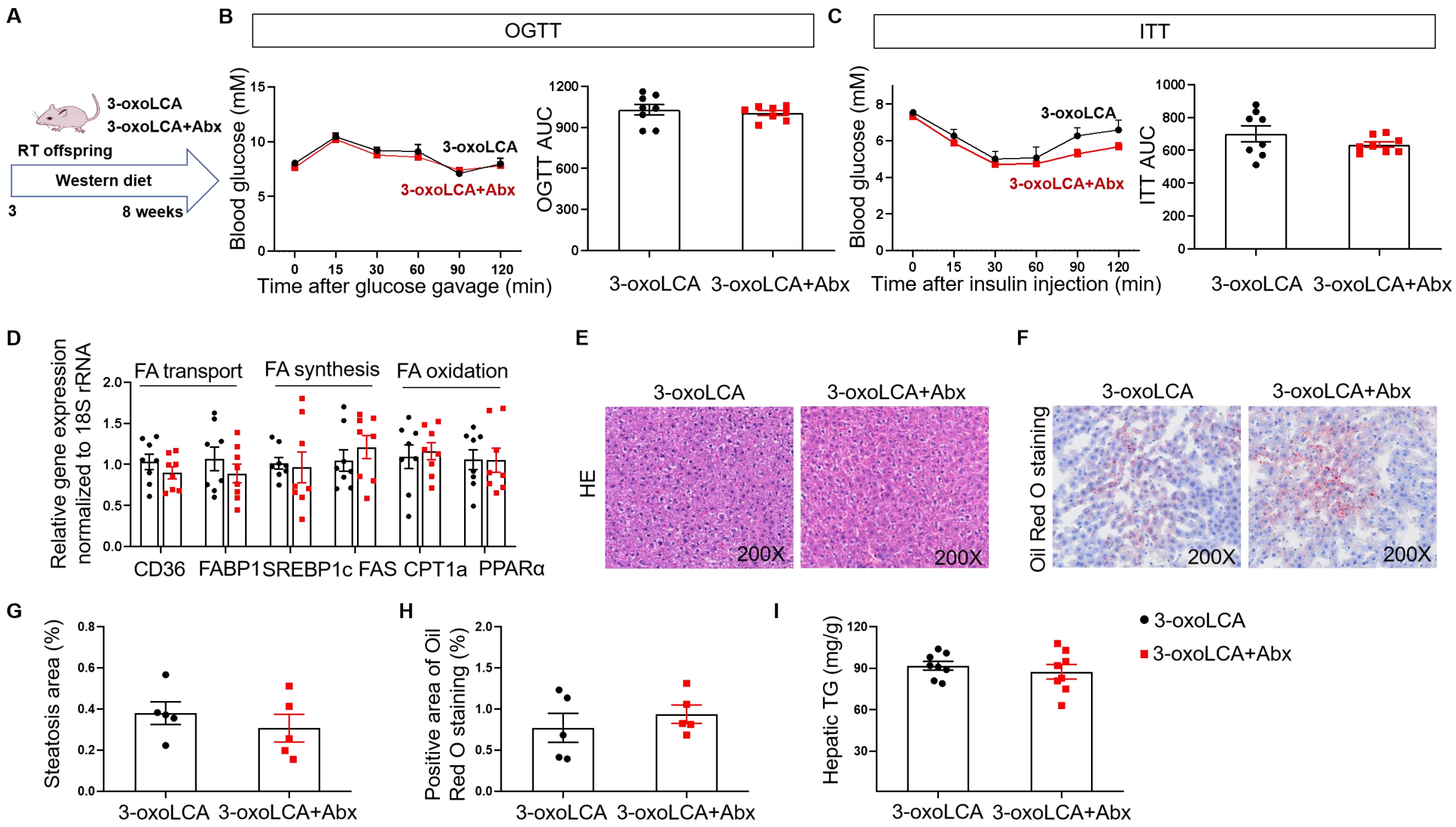

Supplementary Figure 8. Antibiotic treatment does not affect the metabolic protection conferred by 3-oxo-LCA in RT offspring under Western diet challenge.

(A) Experimental design. RT offspring were administered 3-oxo-LCA alone or in combination with antibiotics (3-oxo-LCA+Abx) from 3 to 8 weeks of age while maintained on a Western diet.

(B–C) Glucose and insulin tolerance tests. (B) Oral glucose tolerance test (OGTT) and area under the curve (AUC) analysis. (C) Insulin tolerance test (ITT) and AUC analysis.

(D) Relative hepatic gene expression of fatty acid transport (CD36, FABP1), lipogenesis (SREBP1c, FAS), and β-oxidation (CPT1α, PPARα) in 3-oxo-LCA and 3-oxo-LCA+Abx groups (n = 8 per group).

(E–F) Representative liver histology. (E) H&E staining and (F) Oil Red O staining of hepatic lipid droplets at 200× magnification.

(G–I) Quantification of hepatic lipid accumulation. (G) Steatosis area from H&E staining, (H) Oil Red O-positive area, and (I) hepatic triglyceride (TG) content.

Data are presented as mean ± SEM and compared by Student's t test.

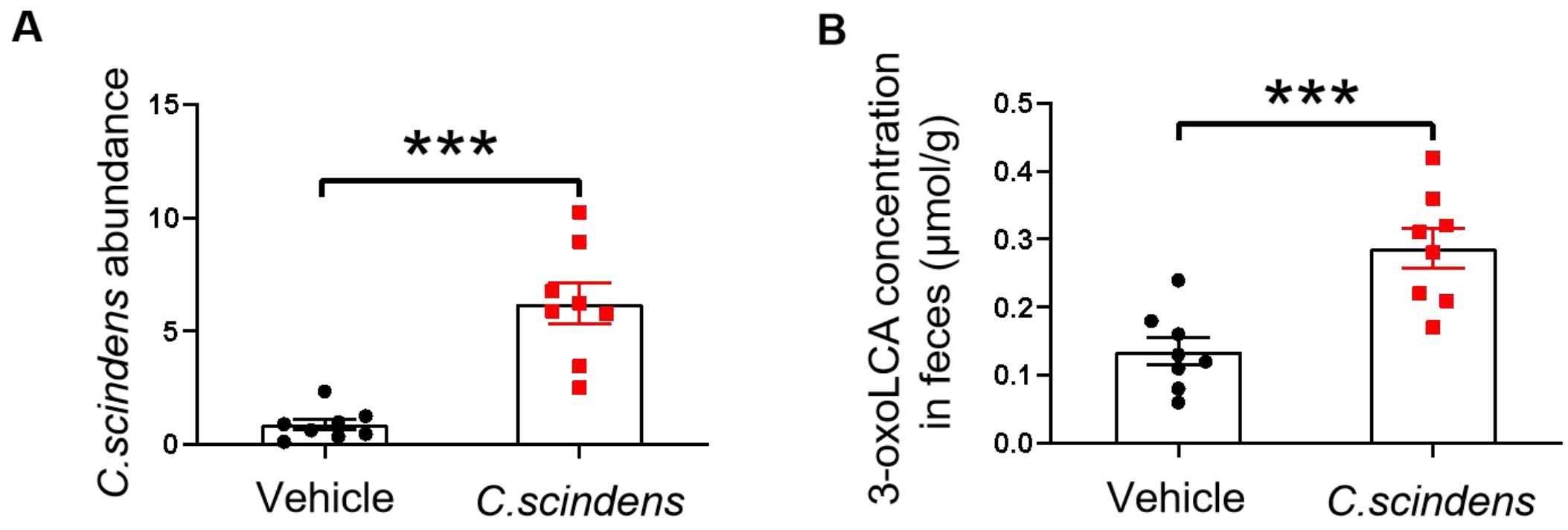

Supplementary Figure 9. Supplementation with *Clostridium scindens* increases its abundance and fecal 3-oxo-LCA levels in RT offspring.

(A) Relative abundance of *C. scindens* in the feces of RT offspring administered vehicle or *C. scindens* (n = 8 per group).

(B) Fecal 3-oxo-LCA concentration in RT offspring treated with vehicle or *C. scindens* (n = 8 per group).

Data are presented as mean ± SEM and compared by Student's t test.
