# Supplementary material for: Maternal cold exposure improves offspring metabolic health via a milk lithocholic acid–microbiota–Th17 axis
Source: NPJ Biofilms Microbiomes. 2026 Jul 9;12:218. doi: 10.1038/s41522-026-01092-7 (PMC13724693; doi:10.1038/s41522-026-01092-7)
Supplement: Supplementary file 2 [file 41522_2026_1092_MOESM2_ESM.pdf]

NOTE: Please save this file locally before filling in the table, DO NOT work on the file within your internet browser as changes will not be saved. Adobe Acrobat Reader (available free here) is recommended for completion.

The ARRIVE guidelines 2.0: author checklist

The ARRIVE Essential 10

These items are the basic minimum to include in a manuscript. Without this information, readers and reviewers cannot assess the reliability of the findings.

Item		Recommendation	Section/line number, or reason for not reporting
**Study design**	1	For each experiment, provide brief details of study design including:	
		a. The groups being compared, including control groups. If no control group has been used, the rationale should be stated.	Manuscript Methods lines 131-223
		b. The experimental unit (e.g. a single animal, litter, or cage of animals).	Manuscript Methods lines 144-153
**Sample size**	2	a. Specify the exact number of experimental units allocated to each group, and the total number in each experiment. Also indicate the total number of animals used.	Manuscript Methods lines 152-153
		b. Explain how the sample size was decided. Provide details of any *a priori* sample size calculation, if done.	Manuscript Methods lines 152-153
**Inclusion and exclusion criteria**	3	a. Describe any criteria used for including and excluding animals (or experimental units) during the experiment, and data points during the analysis. Specify if these criteria were established *a priori*. If no criteria were set, state this explicitly.	Manuscript Methods lines 155-163
		b. For each experimental group, report any animals, experimental units or data points not included in the analysis and explain why. If there were no exclusions, state so.	Manuscript Methods lines 155-156
		c. For each analysis, report the exact value of *n* in each experimental group.	Figure1-Figure6
**Randomisation**	4	a. State whether randomisation was used to allocate experimental units to control and treatment groups. If done, provide the method used to generate the randomisation sequence.	Manuscript 168-173
		b. Describe the strategy used to minimise potential confounders such as the order of treatments and measurements, or animal/cage location. If confounders were not controlled, state this explicitly.	Manuscript Methods lines 172-176
**Blinding**	5	Describe who was aware of the group allocation at the different stages of the experiment (during the allocation, the conduct of the experiment, the outcome assessment, and the data analysis).	Manuscript Methods lines 168-176
**Outcome measures**	6	a. Clearly define all outcome measures assessed (e.g. cell death, molecular markers, or behavioural changes).	Manuscript Methods lines 275-339
		b. For hypothesis-testing studies, specify the primary outcome measure, i.e. the outcome measure that was used to determine the sample size.	Manuscript Methods lines 290-332
**Statistical methods**	7	a. Provide details of the statistical methods used for each analysis, including software used.	Manuscript Statistical analysis 340-350
		b. Describe any methods used to assess whether the data met the assumptions of the statistical approach, and what was done if the assumptions were not met.	Manuscript Statistical analysis 340-350
**Experimental animals**	8	a. Provide species-appropriate details of the animals used, including species, strain and substrain, sex, age or developmental stage, and, if relevant, weight.	Manuscript methods 138-139
		b. Provide further relevant information on the provenance of animals, health/immune status, genetic modification status, genotype, and any previous procedures.	Manuscript methods 139-141
**Experimental procedures**	9	For each experimental group, including controls, describe the procedures in enough detail to allow others to replicate them, including:	
		a. What was done, how it was done and what was used.	Manuscript methods 182-223
		b. When and how often.	Manuscript methods 182-223
		c. Where (including detail of any acclimatisation periods).	Manuscript methods 182-223
		d. Why (provide rationale for procedures).	Manuscript methods 182-223
**Results**	10	For each experiment conducted, including independent replications, report:	
		a. Summary/descriptive statistics for each experimental group, with a measure of variability where applicable (e.g. mean and SD, or median and range).	Figure1, 3,4, 5,6
		b. If applicable, the effect size with a confidence interval.	Figure1-6

The Recommended Set

These items complement the Essential 10 and add important context to the study. Reporting the items in both sets represents best practice.

Item		Recommendation	Section/line number, or reason for not reporting
**Abstract**	11	Provide an accurate summary of the research objectives, animal species, strain and sex, key methods, principal findings, and study conclusions.	manuscript lines 23-43
**Background**	12	a. Include sufficient scientific background to understand the rationale and context for the study, and explain the experimental approach.	manuscript lines 23-43
		b. Explain how the animal species and model used address the scientific objectives and, where appropriate, the relevance to human biology.	manuscript lines 23-43
**Objectives**	13	Clearly describe the research question, research objectives and, where appropriate, specific hypotheses being tested.	manuscript lines 25-27
**Ethical statement**	14	Provide the name of the ethical review committee or equivalent that has approved the use of animals in this study, and any relevant licence or protocol numbers (if applicable). If ethical approval was not sought or granted, provide a justification.	manuscript lines 101-112
**Housing and husbandry**	15	Provide details of housing and husbandry conditions, including any environmental enrichment.	manuscript lines 138-149
**Animal care and monitoring**	16	a. Describe any interventions or steps taken in the experimental protocols to reduce pain, suffering and distress.	manuscript lines 164-167
		b. Report any expected or unexpected adverse events.	manuscript lines 164-167
		c. Describe the humane endpoints established for the study, the signs that were monitored and the frequency of monitoring. If the study did not have humane endpoints, state this.	manuscript 100-124
**Interpretation/ scientific implications**	17	a. Interpret the results, taking into account the study objectives and hypotheses, current theory and other relevant studies in the literature.	manuscript lines 351-546
		b. Comment on the study limitations including potential sources of bias, limitations of the animal model, and imprecision associated with the results.	manuscript lines 636-645
**Generalisability/ translation**	18	Comment on whether, and how, the findings of this study are likely to generalise to other species or experimental conditions, including any relevance to human biology (where appropriate).	manuscript lines 629-635
**Protocol registration**	19	Provide a statement indicating whether a protocol (including the research question, key design features, and analysis plan) was prepared before the study, and if and where this protocol was registered.	manuscript lines 100-130
**Data access**	20	Provide a statement describing if and where study data are available.	manuscript lines 656-660
**Declaration of interests**	21	a. Declare any potential conflicts of interest, including financial and non-financial. If none exist, this should be stated.	manuscript lines 670-679
		b. List all funding sources (including grant identifier) and the role of the funder(s) in the design, analysis and reporting of the study.	manuscript lines 431-438
